# Disulfidptosis-based cancer therapy: Opportunity and challenge

**DOI:** 10.1016/j.gendis.2025.102028

**Published:** 2026-01-03

**Authors:** Bo Wang, Zihui Liu, Junjie Li, Jiaxing Xu, Yamei Guo, Jingxue Zhang, Ti Chu, Zhifen Feng, Qiying Jiang, Dongdong Wu

**Affiliations:** a Department of Stomatology, Huaihe Hospital of Henan University, School of Stomatology, Henan University, Kaifeng, Henan 475004, China; b Henan International Joint Laboratory for Nuclear Protein Regulation, School of Basic Medical Sciences, School of Stomatology, Henan University, Kaifeng, Henan 475004, China; c School of Nursing and Health, Henan University, Kaifeng, Henan 475004, China

**Keywords:** Cancer, Disulfidptosis, NADPH, Regulated cell death

## Abstract

Cancer represents a significant global health threat, with traditional treatment modalities often facing substantial limitations. Consequently, novel therapeutic strategies have garnered considerable interest. In recent years, regulated cell death has achieved remarkable success in cancer treatment, as it is crucial for eliminating abnormal cells. Strategies aimed at inducing regulated cell death, especially disulfidptosis, have become increasingly important in cancer therapy. As a newly identified form of cell death, disulfidptosis has attracted growing attention in cancer research. This process is intricately linked to cystine uptake, consumption, and alterations in the actin cytoskeleton. In addition, disulfidptosis has been effectively utilized in bioinformatics research and has emerged as a biomarker for various cancer types, with the potential to serve as a therapeutic target. This review provides a comprehensive analysis of the canonical disulfidptosis signaling pathway, recent research advancements, and its application potential in oncology. We also examine current bioinformatics-derived biomarkers and potential targets. Crucially, we explore potential research directions for disulfidptosis in cancer and identify existing research challenges.

## Introduction

Cancer remains a major global health burden, accounting for approximately 10 million deaths and 20 million new cases in 2022, with both incidence and mortality on the rise.^1^ Standard-of-care approaches—surgical resection, ionizing radiation, cytotoxic chemotherapy, and immunotherapy—are commonly used in combination yet have inherent limitations. This review puts forward a novel therapeutic model that incorporates disulfidptosis.^2^^,^^3^ Disulfidptosis possesses distinct mechanisms and potential therapeutic targets across various cancer types. This review aims to provide an in-depth analysis of the role of disulfidptosis in cancer, with a particular focus on recent research progress. Moreover, we address current challenges in disulfidptosis research and propose future directions, thereby providing insights and references for subsequent studies. Emerging therapies, including targeted therapy and immunotherapy, have shown considerable potential in cancer treatment.^4^ Programmed cell death pathways—autophagy, pyroptosis, ferroptosis, and necroptosis—have become viable targets for both precision therapies and immunomodulatory strategies.^5^ Pharmacological regulation of apoptosis-regulatory circuits is anticipated to produce novel interventions for refractory malignancies, with these agents acting synergistically with conventional cytotoxic chemotherapy and ionizing radiation.

Disulfidptosis represents a newly identified and distinct form of cell death.^6^ Similar to other regulated cell death modalities—such as autophagy, pyroptosis, ferroptosis, and necroptosis—disulfidptosis acts as a key factor in neoplastic metabolic therapy.^7^ Consequently, its study is gaining importance in oncological research. By utilizing the mechanistic features of disulfidptosis, therapeutic innovations can be achieved via pharmacological inhibition of glucose transporters (GLUTs) or the cystine importer solute carrier family 7 member 11 (SLC7A11), thereby inducing disulfidptosis-mediated tumor suppression.^8^ This cascade is closely linked to the intracellular redox environment, especially within the tumor microenvironment, where redox imbalance often impairs neoplastic viability and proliferative capacity.^9^^,^^10^

Empirical evidence confirms strong correlations between disulfidptosis and oncogenesis. Integrative analysis of disulfidptosis-associated gene expression profiles and mutational spectra has identified distinct molecular subtypes. This analysis also clarifies their mechanistic contributions to tumor progression.^11^ Furthermore, disulfidptosis-centered studies provide novel insights for precision oncology, particularly in terms of early detection and prognostic stratification of high-mortality malignancies such as ovarian and breast carcinomas.^12^^,^^13^

Subsequent studies have demonstrated that disulfidptosis regulates not only the survival of neoplastic cells but also the tumor microenvironment by altering immune-infiltration dynamics.^14^ Specifically, disulfidptosis-related gene (DRG) expression signatures exhibit strong correlations with clinical prognosis and identify actionable nodes for therapeutic intervention.^15^ Consequently, a mechanistic understanding of disulfidptosis is essential for comprehensively characterizing cancer biology and for the rational development of innovative oncological therapies.

In summary, rigorous research on disulfidptosis in oncogenesis is essential for enhancing our mechanistic understanding of tumor biology and facilitating the development of innovative therapeutic models. Sustained advancements in this field is expected to bring about measurable improvements in patient survival and quality of life.^16^ This review systematically outlines the molecular basis of disulfidptosis, assesses its translational applications in neoplastic therapy, and integrates the latest experimental and clinical advances, thereby providing a foundational framework for future disulfidptosis-focused research and clinical practice.

## Disulfidptosis

### Overview of disulfidptosis

In humans, various cell types activate self-destruction mechanisms to maintain systemic homeostasis and counteract pathogenic threats. This evolutionarily conserved process, termed regulated cell death, plays an indispensable role in biological functions such as the elimination of aberrant cells.^17^ Disulfidptosis, a newly characterized regulated cell death modality, emerges from nutrient stress-induced oncogenic reprogramming. Specifically, glucose deprivation drives pathological cystine accumulation in malignant cells overexpressing the cystine transporter SLC7A11. This metabolic perturbation induces pathological disulfide cross-linking within F-actin filaments, leading to cytoskeletal hypercontraction, plasma membrane rupture, and irreversible cell death.^18^ While mechanistic insights remain preliminary, emerging evidence indicates functional crosstalk between disulfidptosis and canonical cell death pathways, including apoptosis, necrosis, and autophagy, suggesting an interconnected cell death regulatory network.^19^, ^20^, ^21^

Disulfidptosis was first described in 2023 by Liu et al through a systematic investigation of metabolic vulnerability in cancer models. In their study, the authors examined the effects of glucose deficiency on cells with elevated SLC7A11 expression, including UMRC6, H460, A549, and 786-O lines. Under glucose deprivation conditions, SLC7A11-high UOK-262 renal carcinoma cells exhibited extensive cell death within 12 h, with mortality reaching approximately 80%. In contrast, mortality was significantly lower (< 20%) in both the control groups and the SLC7A11-knockout cells. Notably, neither iron chelation (DFO) nor pathway-specific inhibitors targeting ferroptosis (ferrostatin-1), apoptosis (Z-VAD-fmk), necroptosis (Nec-1/2), or autophagy (chloroquine) attenuated the observed cell death phenotype. The results indicated that these inhibitors failed to rescue glucose starvation-induced cell death in SLC7A11-overexpressing cells. To further validate their findings, Liu et al examined cells deficient in acyl-CoA synthetase long chain family member 4 (ACSL4), an enzyme critical for ferroptosis, as well as cells lacking bcl-2-associated x protein (BAX) and bcl-2 antagonist/killer (BAK), key regulators of apoptosis, and observed consistent results. However, supplementation with reducing agents that reduce disulfide stress, such as dithiothreitol (DTT), 2-mercaptoethanol, and tris(2-carboxyethyl)phosphine, eliminated this form of cell death.^22^ In contrast, thiol oxidants—such as diamide and diethyl maleate—significantly enhanced cell lethality. Based on these findings, Liu et al formally named this phenomenon disulfidptosis. Using bio-orthogonal chemistry-based proteomic analysis, the research team identified 90 cysteine sites in SLC7A11-high cells that exhibited a significant increase (≥ 1.5-fold) in disulfide bonding under glucose starvation, including multiple actin cytoskeleton-associated proteins. These aberrantly accumulated disulfide bonds specifically disrupt the structural integrity of the actin cytoskeleton, thereby inducing disulfidptosis.^23^^,^^24^ Following mutation of the critical cysteine residue Cys374 to serine in actin (ACTB), cells expressing this mutant form of ACTB maintained F-actin structural integrity under glucose deprivation, and cell mortality was significantly reduced from 80% to 25%.

To clarify the underlying gene regulatory networks, Liu et al performed an unbiased CRISPR-Cas9 screen and identified the Ras-related C3 botulinum toxin substrate 1 (Rac1)–WAVE regulatory complex (WRC) as essential for filopodium formation, which in turn promotes disulfide isomerase activity. The four key genes enhancing disulfide-isomerase function were SLC7A11, solute carrier family 3 member 2 (SLC3A2), ribophorin I (RPN1), and NCK-associated protein 1 (NCKAP1). SLC3A2 encodes the obligate chaperone of SLC7A11, while RPN1 encodes an N-oligosaccharyl-transferase subunit localized in the endoplasmic reticulum. Knockdown of RPN1 partially reverses the disulfide isomerization process, although the precise mechanism remains elusive. Nck-associated protein 1, encoded by NCKAP1, is a component of the WRC. However, the knockout of NCKAP1 counteracts the effect of SLC7A11 knockout on the key disulfide isomerization process. This suggests that cytoskeletal alterations may not be the only mechanism by which disulfide isomerization occurs.^23^

Quantitative analysis of intracellular metabolites via liquid chromatography-mass spectrometry revealed that, following 4 h of glucose deprivation, sensitive cells exhibited a 15-fold increase in intracellular cystine concentration (rising from a baseline of 5 μM to approximately 75 μM). Simultaneously, the level of reduced glutathione (GSH) decreased by more than 60%, and the NADPH/NADP^+^ ratio was reduced by approximately 70%. This metabolic profile collectively indicates that NADPH depletion plays a critical role in the progression of disulfidptosis. Although the seminal work by Liu et al identified NADPH depletion as the pivotal upstream event inhibiting the reduction of cystine to cysteine, the precise biochemical pathways through which accumulated intracellular disulfides, including cystine, directly or indirectly induce aberrant disulfide crosslinking in actin and other proteins were not fully elucidated in the initial studies.^23^ A fundamental question remains unresolved: does this process occur through spontaneous chemical reactions, or does it require the involvement of specific enzymatic facilitators, such as oxidoreductases? Immunofluorescence staining revealed that within 2 h of disulfidptosis induction, phalloidin-labeled F-actin underwent rapid depolymerization, with its fluorescence intensity decreasing by more than 50%, accompanied by the formation of abnormal protein aggregates. This observation indicates that the actin cytoskeleton serves not only as a key target of disulfide stress but also as a critical structural component mediating the process of disulfidptosis. However, the mechanistic basis for the observed specificity—why this stress predominantly disrupts the actin cytoskeletal network rather than other cysteine-rich cellular proteins or organelles—remains incompletely understood.^22^ It remains unclear whether intrinsic cellular mechanisms actively direct or localize this stress towards actin filaments. Moreover, while the established role of actin is well documented, the potential involvement of additional cytoskeletal components or key regulatory proteins in the disulfidptosis cascade requires more comprehensive investigation.^23^ Current research primarily characterizes disulfidptosis as a terminal cell death process. However, its pathophysiological significance may be more complex. Critical unresolved questions include whether disulfidptosis invariably serves as a terminal execution mechanism for cancer cells, or whether cells can recover from initial disulfide stress under specific conditions. Additionally, it remains to be determined whether this stressed state constitutes a critical “point of no return” that potentially facilitates cancer metastasis or therapy resistance. Thus, the full physiological and pathological spectrum of disulfidptosis necessitates further exploration.^22^

As a subtype of regulated cell death, disulfidptosis exhibits both significant differences and connections with previously characterized cell death mechanisms, such as apoptosis and ferroptosis.^7^^,^^25^ Apoptosis, a regulated form of programmed cell death, is characterized by specific morphological changes, including cell membrane blebbing, nuclear condensation, and DNA fragmentation. The primary regulatory mechanism of apoptosis involves a cascade of cysteine-aspartic acid-specific proteases, known as caspases.^26^ Ferroptosis is a distinct non-apoptotic form of cell death driven primarily by iron-dependent lipid peroxide accumulation, and is identified by reduced cell volume and increased mitochondrial membrane density.^27^ while disulfidptosis is mechanistically distinct from apoptosis and ferroptosis, there are areas of functional overlap. For example, changes in intracellular redox balance—closely associated with reactive oxygen species (ROS) production during ferroptosis—may intersect with the initiation of disulfidptosis.^28^ Additionally, disulfidptosis appears to overlap with intracellular signaling pathways that also regulate apoptosis and other cell death modalities.^29^ While disulfidptosis, apoptosis, and ferroptosis operate through distinct lethal mechanisms, their mechanistic crosstalk highlights the complex network underlying regulated cell death. To develop novel cancer treatment strategies, future research should examine the intersections of these mechanisms more closely.

### Molecular mechanisms of disulfidptosis

Cysteine is essential for maintaining redox balance, protein synthesis, catalytic activity, and cellular transport processes within the cell.^30^ To meet the increased demand for antioxidant defense, most cancer cells upregulate the uptake of cystine or cysteine from the extracellular environment via specific transporters. In the highly oxidative extracellular milieu, cysteine is prone to oxidation, forming its dimeric counterpart, cystine.^31^^,^^32^ System Xc^–^ is essential for cystine uptake in most cancer cells. Once inside the cell, cystine is converted to cysteine, in a reaction that requires NADPH.^33^ The structure of system Xc-includes xCT (SLC7A11) as the light chain subunit and CD98hc (SLC3A2) as the heavy chain subunit. SLC7A11 serves as the main functional unit, with SLC3A2 primarily assisting in the transport of light chains and not participating in amino acid transport.^31^ Several studies have revealed that SLC7A11 plays a key role in different forms of programmed cell death and maintains redox equilibrium in cancer cells^34^ (as shown in Fig. 1).

**Figure 1:**
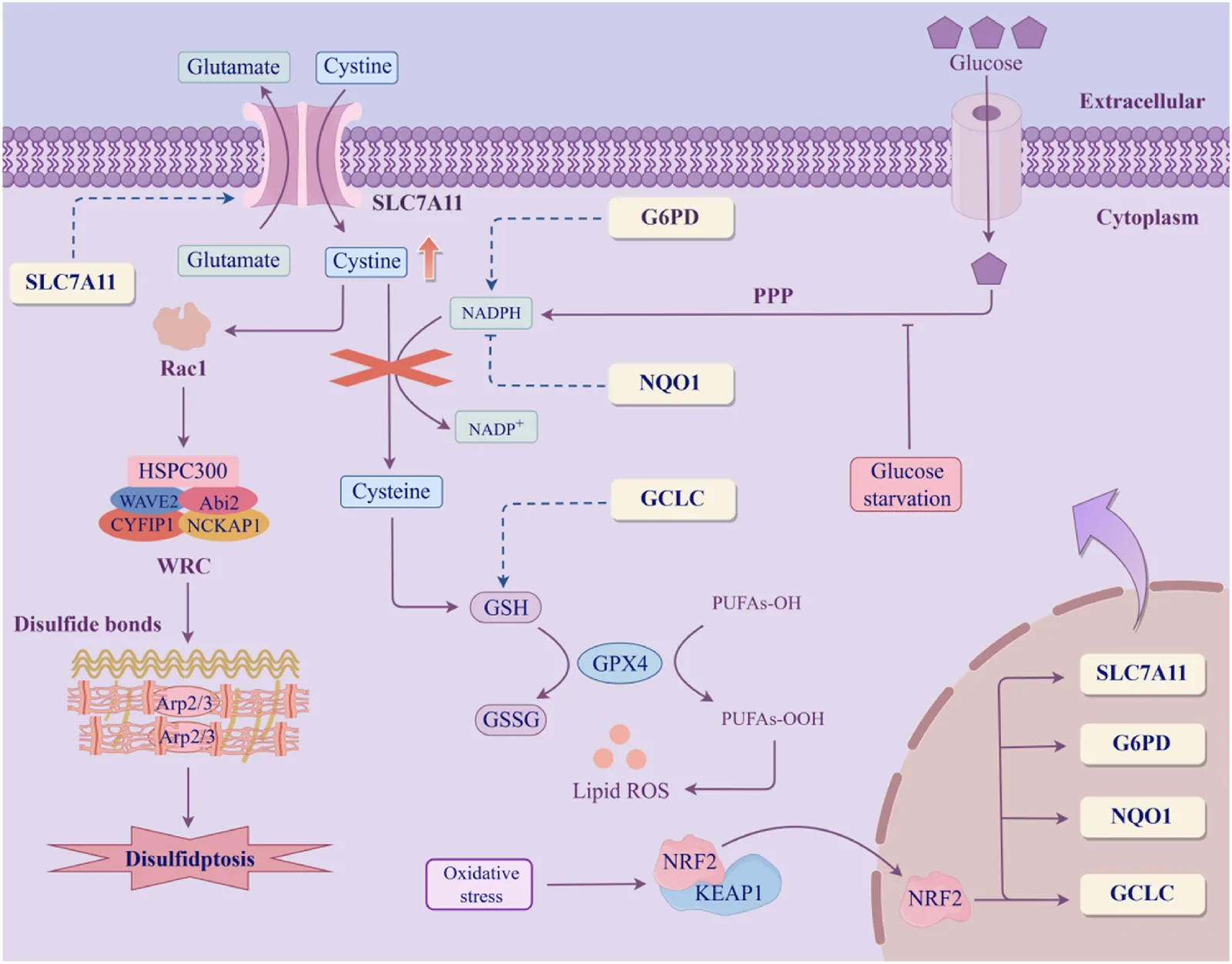
The core mechanism of disulfidptosis. Under conditions of glucose deprivation, a limited supply of NADPH, coupled with elevated cystine uptake in cells exhibiting high SLC7A11 expression, results in the depletion of intracellular NADPH. This scenario leads to the excessive accumulation of cystine and other disulfide-containing molecules, as well as aberrant disulfide bond formation within actin cytoskeletal proteins. Consequently, these processes culminate in the collapse of the actin network and the occurrence of disulfidptosis. The Rac1–WRC-mediated polymerization of branched actin and the subsequent formation of lamellipodia may facilitate the formation of disulfide bonds in actin cytoskeletal proteins, thereby promoting the phenomenon of disulfidptosis. Moreover, oxidative stress can trigger the dissociation of NRF2 from KEAP1. Once activated, NRF2 translocates to the nucleus, where it regulates the expression of downstream target genes via transcriptional mechanisms. Among its core target genes are SLC7A11, G6PD, NQO1, and the catalytic subunit of GCLC. NRF2 plays a bifunctional role in the regulation of disulfidptosis. On one hand, NRF2 enhances cellular cystine uptake by up-regulating SLC7A11 expression. Under conditions of elevated SLC7A11 expression, the simultaneous up-regulation of NQO1 further accelerates NADPH consumption. These combined effects create favorable conditions for the onset of disulfidptosis. Conversely, NRF2 promotes NADPH generation by up-regulating G6PD expression and enhances GSH synthesis through the up-regulation of GCLC. The replenishment of NADPH and the increase in GSH together act to suppress the progression of disulfidptosis. The figure was created by Figdraw. SLC3A2, solute carrier family 3 member 2; SLC7A11, solute carrier family 7a member 11; PPP, pentose phosphate pathway; NADPH, nicotinamide adenine dinucleotide phosphate (reduced form); NADP^+^, nicotinamide adenine dinucleotide phosphate (oxidized form); GSH, glutathione; GSSG, glutathione disulfide; GPX4, glutathione peroxidase 4; ROS, reactive oxygen species; PUFAs–OH, polyunsaturated fatty acids hydroxides; PUFAs–OOH, polyunsaturated fatty acids hydroperoxides; Rac1, ras-related c3 botulinum toxin substrate 1; HSPC300, haematopoietic stem/progenitor cell protein 300; WAVE2, wiskott-aldrich syndrome protein family member 2; Abi2, abelson interactor 2; CYFIP1, cytoplasmic fmr1-interacting protein 1; NCKAP1, nck-associated protein 1; WRC, WAVE regulatory complex; Arp2/3, actin related protein 2/3; NRF2, nuclear factor erythroid 2-related factor 2; KEAP1, Kelch-like ECH-associated protein 1; G6PD, glucose-6-phosphate dehydrogenase; NQO1, NAD(P)H: quinone dehydrogenase 1; GCLC, glutamate–cysteine ligase.

### Non-cancer cells evade disulfidptosis through a tripartite defense mechanism

Non-cancerous cells evade disulfidptosis through a sophisticated tripartite defense mechanism: i) Homeostatic expression of SLC7A11 mitigates pathological cystine overload and subsequent disulfide accumulation;^35^^,^^36^ ii) Adequate glucose flux facilitates the generation of NADPH via the pentose phosphate pathway (PPP), which supplies the necessary reducing equivalents for the conversion of cystine to cysteine and its incorporation into GSH synthesis, thereby preserving redox equilibrium;^35^^,^^37^ iii) The canonical thioredoxin (Trx) and GSH systems continuously monitor and resolve aberrant disulfide bonds. Trx reductase (TrxR) utilizes NADPH to reduce oxidized Trx, which in turn catalyzes the resolution of misformed disulfides in cytosolic proteins, while GSH maintains protein thiol homeostasis through thiol-disulfide exchange reactions.^38^^,^^39^ Collectively, these mechanisms avert pathological actin crosslinking and cytoskeletal collapse, thereby establishing a comprehensive redox buffering system that safeguards normal cells against disulfidptosis under physiological conditions (as shown in Fig. 2).

**Figure 2:**
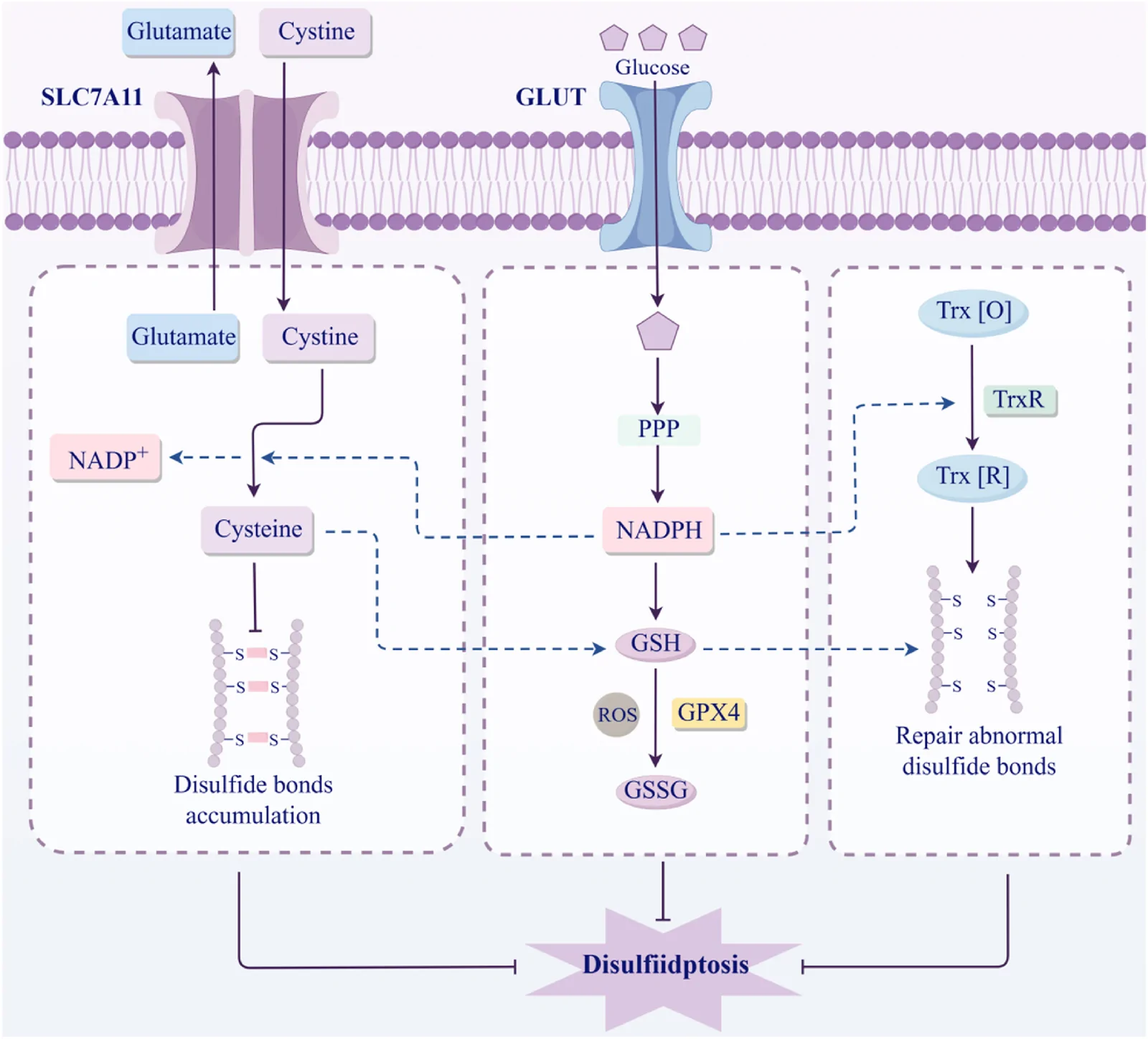
Triple mechanisms of disulfidptosis defense in non-cancer cells. Cells internalize extracellular cystine via the SLC7A11 transporter and depend on GLUT transporters for glucose uptake, which is crucial for initiating metabolic pathways that protect against disulfidptosis. Once glucose enters the PPP, it undergoes enzymatic reactions to produce NADPH. This molecule functions as a cofactor for thioredoxin reductase, facilitating the reduction of oxidized thioredoxin to its reduced form, which directly repairs abnormal disulfide bonds in the actin cytoskeleton, thus preventing their excessive accumulation. Concurrently, NADPH serves as a vital coenzyme for GSH peroxidase, aiding in the reduction of oxidized GSH to its reduced form, thereby enhancing the cell's ability to neutralize reactive oxygen species and maintain redox homeostasis, indirectly inhibiting disulfide bond formation. Additionally, the uptake of cystine via SLC7A11 provides the precursor for cysteine synthesis, supporting GSH production and further bolstering the cellular antioxidant defense system. These pathways operate synergistically, forming an integrated regulatory network that collectively impedes the progression of disulfidptosis. The figure was created by Figdraw. SLC7A11, solute carrier family 7a member 11; PPP, pentose phosphate pathway; NADPH, nicotinamide adenine dinucleotide phosphate (reduced form); NADP^+^, nicotinamide adenine dinucleotide phosphate (oxidized form); GSH, glutathione; GSSG, glutathione disulfide; GPX4, glutathione peroxidase 4; ROS, reactive oxygen species; Trx, thioredoxin; TrxR, thioredoxin reductase.

### Cells with high SLC7A11 expression underwent disulfidptosis under glucose starvation

The transport of cystine plays a pivotal role in the regulation of reduction–oxidation (REDOX) homeostasis. SLC7A11 protects cells against oxidative damage and ferroptosis by mediating cystine import and promoting GSH biosynthesis.^40^ Its expression and activity are regulated by intracellular REDOX status and extracellular signals. In neoplastic settings, increased SLC7A11 expression often reflects enhanced nutrient dependence. Functional studies demonstrate that SLC7A11 modulates antioxidant capacity and interacts with glutaminolysis, thereby influencing glucose–glutamine addiction in malignant cells.^41^ Protein stability is precisely regulated through the CRL3^KCTD10^/E3-USP18/DUB ubiquitination axis, which senses cystine availability to control SLC7A11 turnover and subsequent cystine uptake–ferroptosis dynamics.^42^

Nuclear factor erythroid 2-related factor 2 (NRF2), a key transcription factor regulating cellular redox homeostasis, exerts dual roles within the regulatory network of disulfidptosis.^43^ It mediates the cellular adaptive response to disulfide stress by either directly regulating the PPP or binding to antioxidant response elements (ARE) to transcriptionally activate multiple key target genes, including SLC7A11, glucose-6-phosphate dehydrogenase (G6PD), phosphogluconate dehydrogenase (PGD), glutamate–cysteine ligase (GCLC), and NAD(P)H quinone dehydrogenase 1 (NQO1).^44^

At the molecular level, NRF2 activation can lead to the upregulation of SLC7A11 expression, which, under specific microenvironmental conditions such as nutrient deprivation, may paradoxically enhance intracellular disulfide accumulation, thereby highlighting its context-dependent regulatory role in disulfidptosis.^45^ Clinical studies further emphasize the critical role of the NRF2 pathway in disulfidptosis and its association with drug resistance. In patients with gastric cancer, aberrant activation of the NRF2 pathway is significantly correlated with overexpression of SLC7A11. This patient subgroup exhibits a 42% increase in resistance to 5-fluorouracil chemotherapy, coupled with an approximately 3.2-fold rise in actin disulfide bond modification levels within tumor tissues, indicating the potential role of disulfidptosis in resistance development.^46^ In the context of hepatocellular carcinoma, gaudichaudione H (GH) has been shown to enhance cellular sensitivity to disulfide bond formation through the NRF2–SLC7A11 signaling axis.^43^ Conversely, valproic acid-induced activation of the NRF2–G6PD pathway facilitates the production of NADPH and GSH, thereby inhibiting disulfide crosslinking between cytoskeletal proteins and attenuating disulfidptosis in hepatocellular carcinoma.^47^ Functional validation experiments demonstrate that in NRF2-mutant gastric cancer xenograft mouse models, combined treatment with the GLUT inhibitor WZB117 and the NRF2 inhibitor ML385 effectively induces tumor disulfidptosis, leading to a 65% reduction in tumor volume and the accumulation of cleaved cysteine-modified actin fragments.^47^ Furthermore, in SLC7A11-knockout A549 lung cancer cells, the rate of disulfide stress agent-induced cell death significantly decreases from 78% to 22%. The overexpression of GCLC or NQO1 can partially mitigate the disulfidptosis phenotype by enhancing GSH synthesis and quinone reductase activity, respectively.^48^ Importantly, there exists functional antagonism among NRF2 downstream target genes. For example, while NQO1 overexpression can reduce oxidative damage, in the presence of elevated SLC7A11 expression, it may further deplete NADPH, thereby accelerating disulfidptosis.^45^^,^^48^

These findings not only deepen the understanding of the complex roles of the NRF2 pathway in cell death but also identify new avenues for developing cancer therapeutic strategies targeting disulfidptosis. For instance, combination therapies that target the NRF2/SLC7A11 axis have demonstrated significant tumor-suppressive effects in preclinical models of refractory clear cell renal cell carcinoma, underscoring their considerable translational potential^46^^,^^49^ (as shown in Fig. 1).

SLC7A11 thus functions bidirectionally in REDOX homeostasis: it preserves cellular integrity by promoting GSH biosynthesis while its own expression and activity are reciprocally regulated by intricate signaling networks and microenvironmental cues that ultimately dictate metabolic flux and cell fate. Renal carcinoma cells exhibit elevated SLC7A11 expression, which expedites cytosolic glucose depletion and concomitantly intensifies NADPH consumption.^35^^,^^50^ This results in the accumulation of non-reducible disulfides, triggering disulfide stress and ultimately culminating in disulfidptosis. Disulfides induce toxic cystine disulfide stress accumulation in cells.^51^ In physiological conditions, NADPH furnishes the reducing power crucial for mitigating disulfide bond stress and maintaining cellular homeostasis. Under glucose deprivation, the PPP is impaired in its ability to generate NADPH. Excessive cystine reduces intracellular NADPH levels, leading to NADPH exhaustion and a subsequent accumulation of disulfide molecules. Disulfidptosis initiates with aberrant disulfide bond formation in actin cytoskeletal proteins, leading to destabilization of the F-actin network and subsequent cytoskeletal collapse.^52^

### Actin cytoskeleton changes during disulfidptosis

The actin cytoskeleton is essential for cell migration, adhesion, and morphological stability, with disulfide bond formation serving as a key regulatory mechanism in these processes.^53^ REDOX regulation of actin-binding proteins is crucial for the rapid cellular response. Modifications to oxidation-sensitive cysteine and methionine residues can affect actin cytoskeleton dynamics, thereby influencing cellular migration and adhesion capabilities.^54^

Notably, actin cytoskeleton proteins such as FLNA/B, MYH9, TLN1, and ACTB are preferentially targeted by increased disulfide bond formation in cells with up-regulated SLC7A11 expression.^23^ In these cells, actin cytoskeleton proteins establish multiple intermolecular disulfide bonds, a phenomenon linked to NADPH depletion and independent of ROS. NADPH depletion and disulfide accumulation lead to aberrant actin cytoskeleton dynamics, characterized by F-actin contraction and associated with cell margin retraction and lamellipodial collapse. This process is independent of ROS but dependent on excessive cystine accumulation.^23^

### A key mechanism in the regulation of disulfidptosis is the Rac1–WRC signaling pathway

The WRC pathway plays a crucial role in cell death mechanisms, particularly during disulfidptosis.^39^ Rac1, a Rho-family small GTPase, regulates cytoskeletal dynamics and cellular signaling. Its downstream effector, the WRC, mediates cytoskeletal reorganization and locomotion. Rac1 controls morphological plasticity and motility through WRC-dependent induction of branched actin polymerization.^55^

As a Ras-related small GTPase, Rac1 serves as a key regulatory node in disulfidptosis. This binary molecular switch alternates between an inactive GDP-bound conformation and an active GTP-bound conformation. Conversion to the GTP-bound state is mediated by guanine nucleotide exchange factors (GEFs) that trigger structural rearrangements facilitating nucleotide exchange; inactivation is accelerated by GTPase-activating proteins (GAPs) that enhance intrinsic GTP hydrolysis to restore the GDP-bound state.^56^ The dynamic balance between GEF-mediated activation and GAP-mediated inactivation thus dictates Rac1's functional effects in cellular contexts.

The WRC is a heteropentamer composed of CYFIP1/2, NCKAP1, WAVE1/2/3, HSPC300, and Abi1/2/3. CYFIP1 and NCKAP1 form a dimeric scaffold; WAVE, HSPC300, and Abi constitute a trimeric module. In the basal, autoinhibited state, the verprolin-central-acidic (VCA) domain is sterically occluded by the CYFIP1 concave surface and the WAVE meander segment.^57^ The VCA domain—a conserved structural element in WASP/WAVE family proteins, including WASP, N-WASP, and WAVE/SCAR—acts as the key activator of the actin-related protein 2/3 (Arp2/3) complex, initiating actin nucleation and branched network assembly. Rac1 binds to the A and D sites of CYFIP1 to relieve autoinhibition, releasing the VCA domain; Arf GTPase binding to NCKAP1 further potentiates this activation.^16^

The Rac1–WRC axis mediates disulfidptosis by promoting branched actin polymerization and increasing disulfide bond accumulation within the actin cytoskeleton; experimental silencing of WRC components attenuates this death modality, whereas overexpression of Rac1 or its WRC partners exacerbates cell lethality. A multitude of factors, such as GEFs, GAPs, and guanine nucleotide-dissociation inhibitors (GDIs), are intricately involved in the regulation of Rac1 activity. Furthermore, Rac1's function can be modulated through a variety of post-translational modifications, including phosphorylation and palmitoylation.^16^ Consequently, targeting the Rac1–WRC pathway represents a promising therapeutic strategy for cancer treatment via the induction of disulfidptosis.

### Disulfidptosis versus ferroptosis

Ferroptosis is an iron-dependent form of programmed cell death, characterized by the breakdown of the antioxidant defense system, the accumulation of specific lipid peroxides, and the disruption of iron metabolism.^58^ The principal molecular pathways involved in ferroptosis are centered around the system Xc^−^–GSH–GPX4 axis.^59^ System Xc^−^ imports cystine required for GSH synthesis; GPX4 utilizes GSH to reduce phospholipid hydroperoxides and thereby suppresses lipid peroxidation.^60^ Simultaneously, ACSL4 and lysophosphatidylcholine acyltransferase 3 (LPCAT3) collaborate to promote the synthesis of polyunsaturated fatty acid-containing phospholipids, which are essential substrates for lipid peroxidation.^61^ In the presence of divalent iron ions, Fenton reaction-driven chain reactions lead to the excessive accumulation of membrane lipid peroxides, ultimately impairing plasma membrane integrity and leading to cell death.^62^ Therefore, enhanced cystine uptake and subsequent GSH synthesis facilitate the activation of GPX4, thereby conferring resistance to ferroptosis.^63^ In contrast, erastin impedes cystine uptake, leading to GSH depletion, GPX4 inactivation, and the induction of ferroptosis.^63^ Consequently, cancer cells with enhanced cystine import capacity demonstrate heightened resistance to ferroptosis.

In various cancer models characterized by elevated SLC7A11 expression, while the overexpression of SLC7A11 enhances cellular resistance to ferroptosis, it simultaneously increases susceptibility to glucose deprivation, thereby promoting disulfidptosis.^64^, ^65^, ^66^, ^67^, ^68^, ^69^ Under glucose-deficient conditions, the sustained influx of cystine mediated by SLC7A11 cannot be efficiently reduced to cysteine due to the depletion of NADPH, resulting in the abnormal intracellular accumulation of cystine and other disulfides, which triggers disulfide stress.^22^ Concurrently, the activation of the Rac1–WRC–Arp2/3 signaling pathway promotes the formation of aberrant disulfide bonds in actin filaments, leading to the aggregation of the F-actin network and cytoskeletal collapse, ultimately culminating in cell death.^23^ The specific mechanisms underlying this process have been previously discussed and will not be reiterated here. However, it is evident that the capacity for cystine uptake closely links disulfidptosis with ferroptosis, yet the two processes differ significantly in target specificity, metabolic triggers, molecular pathways, and pharmacological specificity (as shown in Fig. 3).

**Figure 3:**
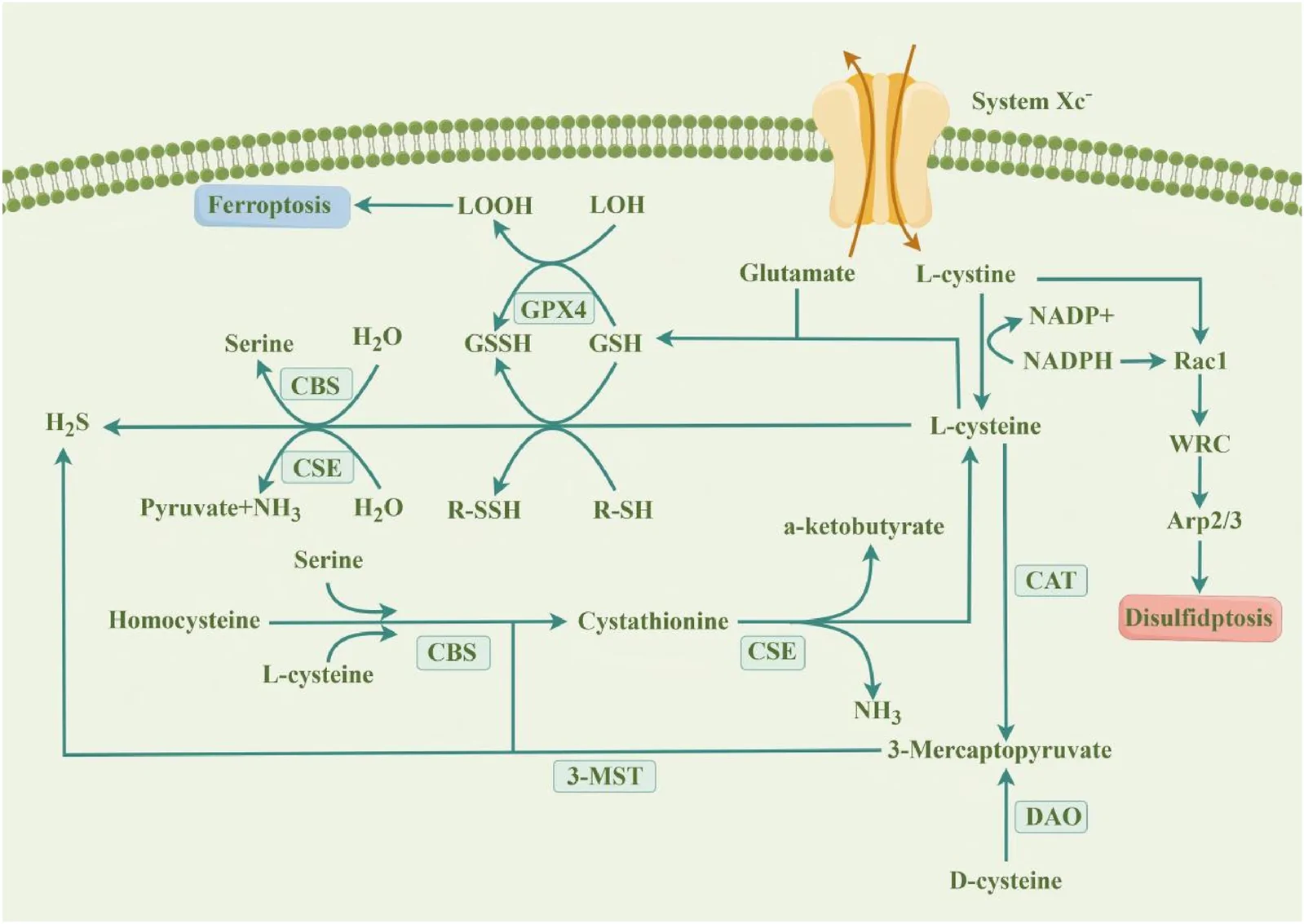
The link between sulfide metabolites and ferroptosis and disulfidptosis. Cysteine serves as a crucial intermediate in intracellular sulfur metabolism. It is synthesized to generate H_2_S via the catalytic actions of CSE and CBS, or through pathways involving CAT and 3-MST. Furthermore, cysteine metabolism is intricately linked to cellular processes such as disulfidptosis and ferroptosis. The accumulation of cystine can lead to disulfidptosis, whereas GSH, synthesized from cysteine, can reduce lipid hydroperoxides to lipid alcohols through the activity of GPX4, thereby inhibiting ferroptosis. The figure was created by Figdraw. H_2_S, hydrogen sulfide; CSE, cystathionine-γ-lyase; CBS, cystathionine-β-synthase; CAT, cysteine aminotransferase; 3-MST, 3-mercaptopyruvate sulfurtransferase; GPX4, glutathione peroxidase 4; GSSH, glutathione disulfide; GSH, glutathione; LOH, lipid alcohol; LOOH, lipid hydroperoxide; NADPH, nicotinamide adenine dinucleotide phosphate (reduced form); NADP^+^, nicotinamide adenine dinucleotide phosphate (oxidized form); Rac1, Ras-related c3 botulinum toxin substrate 1; WRC, WAVE regulatory complex; Arp2/3, actin related protein 2/3; DAO, D-amino acid oxidase.

At the level of mechanistic specificity, ferroptosis and disulfidptosis exhibit fundamental differences. Ferroptosis primarily targets membrane phospholipids and is driven by iron-dependent lipid peroxidation, whereas disulfidptosis specifically targets cytoskeletal proteins and is mediated by aberrant disulfide crosslinking within the actin network.^37^^,^^70^ In terms of metabolic triggers, ferroptosis is typically induced by the inactivation of GPX4 or the depletion of GSH.^71^ In contrast, disulfidptosis is strictly dependent on NADPH depletion and cystine accumulation, resulting from the combination of high SLC7A11 expression and glucose deprivation.^23^ In terms of molecular pathways, ferroptosis is regulated by the GPX4–GSH system and the FSP1–CoQ10–NAD(P)H axis, along with other lipid peroxidation defense mechanisms.^72^^,^^73^ In contrast, disulfidptosis is closely associated with the Rac1–WRC–Arp2/3 pathway, which mediates dynamic cytoskeletal reorganization.^16^ The two forms of cell death also differ significantly in pharmacological responses: ferroptosis can be specifically inhibited by the lipid antioxidant Ferrostatin-1, yet it remains unresponsive to DTT; conversely, disulfidptosis can be effectively reversed by DTT but is unaffected by Ferrostatin-1.^74^^,^^75^ A comprehensive understanding of the fundamental mechanistic differences between these two cell death modalities is essential for the rational design of combination therapeutic strategies and the co-targeting of these distinct processes. However, to date, no mature preclinical models or clinical studies have been publicly reported that directly combine ferroptosis and disulfidptosis induction. Consequently, their synergistic potential and translational pathways require further systematic investigation (Table 1).

**Table 1:** The difference between disulfidptosis and ferroptosis.

Comparison dimension	Ferroptosis	Disulfidptosis
Target	Primarily targets membrane phospholipids and is driven by iron-dependent lipid peroxidation	Specifically targets cytoskeletal proteins and is mediated by aberrant disulfide crosslinking within the actin network
Metabolic triggers	Typically induced by the inactivation of GPX4 or depletion of GSH	Strictly depends on NADPH depletion and cystine accumulation, resulting from the combination of high SLC7A11 expression and glucose deprivation
Molecular pathways	Regulated by lipid peroxidation defense mechanisms such as the GPX4–GSH system and the FSP1–CoQ10–NAD(P)H axis	Closely linked to the Rac1–WRC–Arp2/3 pathway, which mediates dynamic cytoskeletal reorganization
Pharmacological responses	Specifically inhibited by the lipid antioxidant ferrostatin-1, but is unresponsive to DTT	Effectively reversed by DTT but is unaffected by ferrostatin-1

Note: GPX4, glutathione peroxidase 4; GSH, glutathione; DTT, dithiothreitol; NADPH, nicotinamide adenine dinucleotide phosphate; SLC7A11, solute carrier family 7 member 11; FSP1, ferroptosis suppressor protein 1; WRC, WAVE regulatory complex; CoQ10, coenzyme Q10; Rac1, ras-related C3 botulinum toxin substrate 1; Arp2/3, actin-related protein 2/3.

## Disulfidptosis and cancer

### Disulfidptosis and bioinformatics

The discovery of disulfidptosis stemmed from meticulously designed experiments; however, the comprehensive elucidation of its regulatory network has predominantly depended on the advanced analytical capabilities of bioinformatics.^76^ By utilizing extensive public omics datasets, researchers swiftly constructed a “genetic landscape” of disulfidptosis and clarified its complex interactions with other vital cellular processes.^77^

The study conducted by Liu et al identified the involvement of several core genes, including SLC3A2, in the initiation of disulfidptosis.^23^ Nevertheless, a complete biological process inherently involves a complex gene regulatory network. The initial step in bioinformatic analysis is the systematic identification of all critical components within this network, specifically the DRGs.^78^ Researchers commenced by compiling a list of known genes associated with disulfide stress and the actin cytoskeleton from existing literature, thereby establishing an initial set of “seed genes”.^66^^,^^79^ Subsequently, they performed large-scale transcriptomic data mining using resources such as the Cancer Genome Atlas (TCGA) and the Genotype-Tissue Expression Project.^66^^,^^80^ Through comprehensive data acquisition and integration, differential expression analysis, and assessments of genetic and epigenetic variations, the disulfidptosis gene set was expanded from a small number of initial members to a complex network comprising dozens of core components. These components include actin cytoskeletal proteins (*e.g.*, ACTN4, FLNA, MYH9), nucleoporins (*e.g.*, NUBPL, RPN1), and proteins related to the mitochondrial respiratory chain (*e.g.*, NDUFA11, NDUFS1, LRPPRC), among others.^79^^,^^81^ Following the identification of DRGs, functional enrichment analyses using bioinformatics approaches allowed researchers to elucidate the biological context in which these genes operate cooperatively. By employing Gene Ontology and Kyoto Encyclopedia of Genes and Genomes enrichment analyses, gene set variation analysis, and network biology, disulfidptosis was contextualized within an extensive cellular signaling network, rather than being perceived as an isolated cell death event. This integrative approach enhanced the systematic understanding of its molecular mechanisms.^82^^,^^83^

In the realm of upstream basic research, the dissection of molecular networks epitomizes the contribution of bioinformatics. However, its role in downstream clinical translational research is even more pronounced and direct. Shortly after the introduction of the concept of disulfidptosis, numerous bioinformatics-driven clinical studies emerged, swiftly evaluating its potential as a prognostic biomarker and therapeutic target.

The initial phase of translational research involves evaluating the clinical prognostic significance of DRGs, a process for which bioinformatics offers a standardized and efficient analytical framework. Through a comprehensive series of rigorous bioinformatic analyses, including the identification of prognostic genes, development of risk scoring models, evaluation and validation of model performance, independent external validation, and the construction of clinical nomograms, risk models with significant prognostic value related to disulfidptosis have been successfully developed for various cancers, including liver cancer, colorectal cancer, renal cancer, and glioma 84, 85, 86, 87, 88, 89 (Table 2). Beyond basic prognostic assessment, the risk prediction models developed in various studies have demonstrated significant success in elucidating tumor heterogeneity, facilitating precise stratified therapy, evaluating the tumor immune microenvironment, and forecasting drug sensitivity.^90^^,^^91^ In a study focusing on pancreatic cancer, unsupervised clustering of TCGA and GEO data effectively identified two molecular subtypes. The C1 subtype, characterized by high expression of cytoskeleton-related DRGs such as FLNA and MYH9, exhibited features of an immunosuppressive microenvironment and showed significantly greater sensitivity to gemcitabine compared with the C2 subtype. Conversely, the C2 subtype, marked by high expression of metabolism-related DRGs such as glycogen synthase 1 (GYS1) and NADH dehydrogenase (ubiquinone) Fe–S protein 1 (NDUFS1), was distinguished by increased CD8^+^ T cell infiltration and a lower TIDE score, indicating its potential as a beneficiary population for immune checkpoint inhibitors.^84^ In the context of clear cell renal cell carcinoma, a DRG-based risk model integrated with spatial multi-omics analysis, revealed specific high expression of the SLC7A11 protein at the tumor periphery in high-risk patients. Single-cell sequencing further identified an enrichment of an ACTB^+^/SLC7A11^+^ cell subpopulation within the tumor core that is susceptible to disulfidptosis. This discovery has directly influenced clinical decision-making, recommending first-line sunitinib targeted therapy for patients classified as low-risk, while suggesting a combination immunotherapy regimen of nivolumab and ipilimumab for those identified as high-risk.^92^^,^^93^ In the context of hepatocellular carcinoma, analysis of the immune microenvironment using CIBERSORT deconvolution indicated that the high-risk DRG group is significantly associated with increased infiltration of M2 macrophages and elevated expression of PD-L1. This finding provides a theoretical foundation for combination therapies involving PD-1 inhibitors and glucose transporter inhibitors to induce disulfidptosis.^94^^,^^95^ Collectively, these examples demonstrate that bioinformatics-driven DRG models are transitioning from tools for prognostic assessment to systems that support treatment decision-making. Their primary value lies in translating molecular heterogeneity into actionable clinical stratification strategies.

**Table 2:** Composition of risk prediction models associated with disulfidptosis in different cancer types.

Cancer types	Risk prediction model related genes	Reference
Glioma	ABI3, APOBEC3C, CD53, RNASE6, OLFML3, HLA-DRB1, TYROBP, SIGLEC10, TNFAIP8L2	86
Cervical cancer	ZSCAN16-AS1, AC083799.1, AL021707.6, LINC02356, AC023043.1	210
Colon adenocarcinoma	AC007728.3, AP003555.1, ATP2B1.AS1, NSMCE1.DT	87
Lung squamous cell carcinoma	AC007728.3, AP003555.1, ATP2B1.AS1, NSMCE1.DT	211
Lung adenocarcinoma	LINC01311, AC010285.3, C10orf55, AL021368.3, AC009509.4, AL606489.1, AL133410.1, AC130462.2, AC005618.4	212
Breast cancer	AL365181.2, AL 606489.1, SNHG12, GSEC, AC090559.1	213
Hepatocellular carcinoma	SLCO1B1, CD5, SLCO1B1, CD79A, ETV5, FZD7, SNX7, SLC1A7	88
Renal cell carcinoma	GATA3-AS1, LINC00511, LINC01488, LINC02188	89
Colorectal cancer	SLCO1B1, CD5, SLCO1B1, CD79A, ETV5, FZD7, SNX7, SLC1A7	214
Sarcoma	SPINT1-AS1, AL121944.1, AC131009.3, AC104088.3, AL035071.1, LINC00886, AL035587.2, AC007743.1	215
Gastric cancer	NDUFS1, NDUFS2, NDUFC1, EPAS1, SLC7A11, OXSM, LRPPRC, NDIFA11, NUBPL, CONT1	216
Ovarian cancer	SLC7A11, RPN1, GYS1	217
Prostate cancer	GLA, HIF-1α, VPS35, CDC37	218
Papillary thyroid carcinoma	CTB-171A8.1, CTD-2371O3.2, LINC00240, RP11-126K1.6, RP11-872J21.3, RP3-500L14.2, SNHG10, TLR8-AS1	219
Pancreatic cancer	MYADM, MPDZ, LTBP2, PAPSS2, DENND4C, PROK1, DDIT4, IGFBP3, CFD, MMP7, PIGY	177
Stomach adenocarcinoma	SNAI1, STC1, PKHD1L1, ANKRD37	220
Head and neck squamous carcinoma	MET, ANLN, ANXA3, NT5E, IGF2BP3, FAM83A, RTN1, AREG, DSG3, KRT6A	221
Uterine corpus endometrial carcinoma	IL10RB-DT, AL139147.1	222
Diffuse large B-cell lymphoma	AC104794.3, AL109936.2	223
Endometrial cancer	AC090617.5, AC007528.1, AC010201.3, NBAT1, XPC-AS1, PRDX6-AS1, AC009779.2, AL445231.1, U91328.1, AC244517.7, BOLA3-AS1, MKLN1-AS, AC093382.1, EIF3J-DT	224
Laryngeal squamous cell carcinoma	CAPZB, DALG, GYS1, IQGAP1, MYH9, NDUFA11, NDUFS1, OXSM	225
Skin cutaneous melanoma	AC005034.2, AC020765.2, AL158071.4, AL161663.2, AP000787.1, CR392039.3, EMSLR, SEC24B-AS1, Z69733.1, Z94721.3	226
Esophageal squamous cell carcinoma	AP003721.3, AC040977.1, ODC1-DT, AL358334.2, AC023310.4	227

Note: ABI3, abi gene family member 3; APOBEC3C, apolipoprotein b mRNA editing enzyme catalytic polypeptide-like 3c; CD53/5/79A, cluster of differentiation 53/5/79A; RNASE6, ribonuclease a family member 6; OLFML3, olfactomedin-like 3; HLA-DRB1, human leukocyte antigen–dr beta chain 1; TYROBP, TYRO protein tyrosine kinase binding protein; SIGLEC10, sialic acid binding ig like lectin 10; TNFAIP8L2, tumor necrosis factor, alpha-induced protein 8-like 2; ZSCAN16-AS1, ZSCAN16 antisense RNA 1; ATP2B1.AS1, ATP2B1 antisense RNA 1; NSMCE1.DT, NSMCE1 downstream transcript; GATA3-AS1, GATA3 antisense RNA 1; SLCO1B1, solute carrier organic anion transporter family member 1b1; ETV5, ETS variant transcription factor 5; FZD7, frizzled receptor 7; SNX7, sorting nexin 7; SLC1A7, solute carrier family 1 member 7; NDUFS1, NADH dehydrogenase (ubiquinone) Fe–S protein 1; NDUFA11, NADH dehydrogenase (ubiquinone) 1 alpha subcomplex subunit 11; NUBPL, nucleotide-binding protein-pike; GYS1, glycogen synthase 1; RPN1, ribophorin I; SLC7A11, solute carrier family 7 member 11; GLA, α-galactosidase A; HIF-1α, hypoxia-inducible factor 1 alpha; VPS35, vacuolar protein sorting 35; CDC37, cell division cycle 37; SNHG10, small nucleolar RNA host gene 10; TLR8-AS1, toll-like receptor 8 antisense RNA 1; MYADM, myeloid-associated differentiation marker; MPDZ, membrane protein, palmitoylated 5; LTBP2, latent transforming growth factor beta binding protein 2; PAPSS2, 3′-phosphoadenosine 5′-phosphosulfate synthase 2; DENND4C, DENN domain containing 4C; PROK1, prokineticin 1; DDIT4, deoxyribonucleic acid damage-inducible transcript 4; IGFBP3, insulin-like growth factor binding protein 3; CFD, complement factor D; MMP7, matrix metalloproteinase 7; PIGY, phosphatidylinositol glycan anchor biosynthesis, class Y; MET, mesenchymal epithelial transition factor; ANLN, anillin; ANXA3, annexin a3; NT5E, 5′-nucleotidase, ecto; IGF2BP3, insulin-like growth factor 2 mRNA binding protein 3; FAM83A, family with sequence similarity 83 member A; RTN1, reticulon 1; AREG, amphiregulin; DSG3, desmoglein 3; KRT6A, keratin 6a; IL10RB-DT, interleukin 10 receptor subunit beta-downstream transcript; NBAT1, non-brain antigen transcript 1; XPC-AS1, xeroderma pigmentosum group c antisense RNA 1; PRDX6-AS1, peroxiredoxin 6 antisense RNA 1.

Despite advancements in bioinformatics, significant limitations persist, primarily due to the disconnect between model predictions and actual biological processes. Current prognostic models predominantly utilize bulk transcriptomic data, which inadequately capture the spatial distribution and dynamic alterations of specific cellular subpopulations within the tumor microenvironment. The intra-tumoral heterogeneity uncovered by single-cell sequencing—such as the observation that only 15%–30% of cells in renal carcinoma exhibit high expression of core DRGs—suggests that existing risk scores may substantially underestimate the impact of critical cellular subsets.^96^ Furthermore, the absence of real-time monitoring technologies for disulfide stress results in delays in mechanistic validation, as current methodologies rely on static tissue sections or endpoint survival analyses to indirectly infer the occurrence of disulfidptosis.^97^ Future research endeavors should prioritize the integration of high-resolution spatial transcriptomics with single-cell sequencing to accurately delineate the spatial architecture and temporal progression of critical cellular subpopulations in solid tumors, such as renal cancer. This approach aims to enhance existing risk models that currently underestimate the heterogeneity of the tumor microenvironment.^96^^,^^97^ Concurrently, the advancement of live-cell imaging and biosensor technologies will be crucial for the dynamic monitoring of key biological processes, such as disulfide stress, thereby surpassing the limitations of static histological analyses.^97^^,^^98^ Ultimately, the development of multimodal prognostic models that synthesize spatial single-cell data, real-time stress indicators, and clinical information—validated through extensive prospective cohort studies—will be imperative.^97^^,^^99^ This strategic pathway, which capitalizes on contemporary technological advancements and algorithmic refinements, offers significant potential for bridging the gap between mechanistic discoveries and clinical decision-making (as shown in Fig. 4).

**Figure 4:**
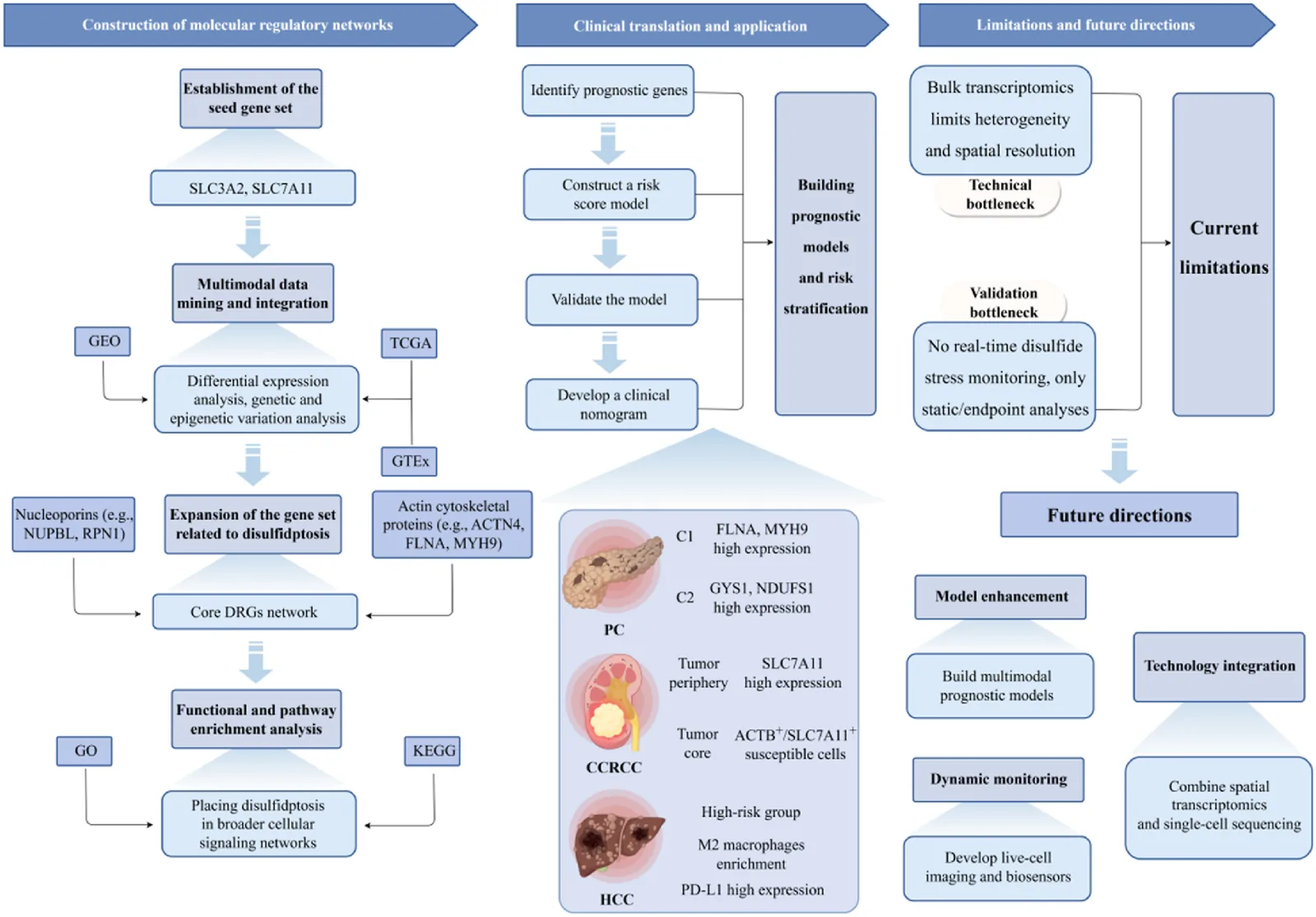
Disulfidptosis and bioinformatics. This figure systematically delineates a comprehensive research framework encompassing the entire spectrum from upstream fundamental mechanism discovery, through midstream clinical translation and application, to downstream limitations and future directions. The upstream phase of fundamental research initiates with experimental findings, employing core genes such as SLC3A2 as the analytical foundation. This phase adheres to a core bioinformatic workflow that includes “seed gene set establishment, multi-omics data mining and integration, DRGs expansion, and functional and pathway enrichment analysis” to construct molecular regulatory networks. The midstream phase of clinical translation and application utilizes a standardized analytical pipeline involving prognostic gene identification, risk score model construction, model validation, and clinical nomogram development to generate disulfidptosis risk scores applicable to liver, pancreatic, and renal cancers. This facilitates the development of prognostic models and risk stratification. The downstream module explicitly outlines the current research limitations and proposes future research directions, thereby comprehensively underscoring the critical supporting role of bioinformatics throughout all stages of disulfidptosis research. The figure was created by Figdraw. SLC3A2, solute carrier family 3 member 2; SLC7A11, solute carrier family 7a member 11; GEO, gene expression omnibus; TCGA, the cancer genome atlas; GTEx, genotype-tissue expression; GO, Gene Ontology; KEGG, Kyoto Encyclopedia of Genes and Genomes; NUBPL, nucleotide binding protein-like; RPN1, ribophorin I; ACTN4, actinin alpha 4; FLNA, filamin A; MYH9, myosin heavy chain 9; CCRCC, clear cell renal cell carcinoma; PC, pancreatic cancer; HCC, hepatocellular carcinoma.

### Cancer therapy based on disulfidptosis

Disulfidptosis, a mode of programmed cell death reliant on the formation and disruption of intracellular disulfide bonds, exhibits distinct biological characteristics. Combining disulfidptosis-inducing agents with treatments like chemotherapy and radiotherapy could greatly improve anti-tumor effectiveness.

### Drugs for treating cancer based on disulfidptosis

Liu et al have explored the anti-cancer effects of inhibitors targeting GLUTs. Their findings demonstrated that both the GLUT1 inhibitor BAY-876 and the GLUT3 inhibitor KL-11743 effectively inhibited glucose uptake in UMRC6 renal cancer cells,^8^ thereby inducing disulfidptosis and suppressing tumor progression. In addition to BAY-876 and KL-11743, recent research has identified further GLUT inhibitors, such as WZB117, STF-31, and 3PO, which induce disulfidptosis by inhibiting glucose uptake.^100^, ^101^, ^102^ However, the translation of fundamental research findings into clinical practice remains fraught with significant challenges, primarily due to the extraordinary metabolic plasticity of cancer cells and the generally inadequate selectivity of current therapeutic agents. Cancer cells can markedly reduce the efficacy of single-target inhibitors by activating various metabolic compensatory mechanisms. For instance, in preclinical ovarian cancer xenograft models, the continuous administration of the GLUT1 inhibitor BAY-876 at a dosage of 4.5 mg/kg/day for two weeks resulted in a significant reduction in tumor volume and weight (approximately 66%–68%)^103^ (Table 3). However, this dosage was associated with some host toxicity. More critically, cancer cells can circumvent the blockade of glucose uptake induced by GLUT1 inhibition by up-regulating alternative GLUTs, such as GLUT2/5 or SGLT1.^103^^,^^104^ Additionally, when the glycolytic pathway is inhibited, cancer cells can sustain their energy homeostasis and survival by enhancing mitochondrial oxidative phosphorylation (OXPHOS) or by shifting to alternative energy sources, such as lactate.^105^

**Table 3:** List of GLUT inhibitors.

Drugs	Molecular architectural features	Reference
Quercetin	C_15_H_10_O_7_	228
	The molecular structure central to the function of C_15_H_10_O_7_ incorporates a catechol moiety (3′,4′-di-OH), a conjugated flavonol scaffold, a C3 hydroxyl group, and a C2=C3 double bond conjugated with the C4 ketone group.	
Fisetin	C_15_H_10_O_6_	229
	The molecular structure central to the function of C_15_H_10_O_6_ incorporates a 3,7-dihydroxychromen-4-one scaffold, a catechol moiety (3′,4′-diOH), a C3–OH group, and distinct protonation sites at C8–H and C6–H.	
WZB117	C_17_H_14_ClN_3_O_3_S	100
	The molecular structure central to the function of C_17_H_14_ClN_3_O_3_S comprises a 4-chlorophenyl group, a sulfonamide bridge (–SO_2_NH–), and a benzothiazole carbonyl moiety (-C=O).	
STF-31	C_17_H_13_N_3_O_3_S_2_	102
	The molecular structure central to the function of C_17_H_13_N_3_O_3_S_2_ comprises a naphthalene group, a sulfonamide linker (–SO_2_NH–), and a benzothiazolone carbonyl moiety (-N-C=O).	
Bay-876	C_18_H_17_N_5_O_3_S	230
	The molecular structure central to the function of C_18_H_17_N_5_O_3_S comprises a 4-chlorophenyl anchor, a sulfonamide bridge (–SO_2_NH–), and a thienopyrimidinone carbonyl moiety (-C=O).	
Phloretin	C_15_H_14_O_5_	231
	The molecular structure central to the function of C_15_H_14_O_5_ comprises a 4′-hydroxyl group, 2′,6′-dihydroxyl pairs, and a carbonyl moiety.	
Sitagliptin	C_16_H_15_F_6_N_5_O	232
	The molecular structure central to the function of C_16_H_15_F_6_N_5_O comprises a 2,4,5-trifluorophenyl group, a β-amino moiety (–CH_2_–NH_2_), and a triazolopiperazine nitrogen motif.	
Tert-butylhydroquinone	C_10_H_14_O_2_	233
	The molecular structure central to the function of C_10_H_14_O_2_ comprises a 4-hydroxyl group, an adjacent phenolic hydroxyl group at position 1, and a tert-butyl substituent.	
Oxaliplatin	C_8_H_14_N_2_O_4_Pt	234
	The molecular structure central to the function of C_8_H_14_N_2_O_4_Pt comprises a Pt(II) center, a DACH hydrophobic ring, and an oxalate bidentate ligand.	
Tunicamycin	C_38_H_64_N_4_O_16_	235
	The molecular structure central to the function of C_38_H_64_N_4_O_16_ comprises a uracil-diphosphate (-UDP) group, an N-acetylglucosamine moiety, and a lipophilic undecaprenyl chain.	
Allicin	C_6_H_10_OS_2_	236
	The molecular structure central to the function of C_6_H_10_OS_2_ comprises a thiosulfinate group (–S(O)S–) and two allyl substituents (-CH_2_CH=CH_2_).	
Lonidamine	C_15_H_10_Cl_2_N_2_O_2_	237
	The molecular structure central to the function of C_15_H_10_Cl_2_N_2_O_2_ comprises an indazole-3-carboxylic acid core, a 4,5-dichlorophenyl group, and a carboxylic acid functional group.	
3PO	C_13_H_10_N_2_O	101
	The molecular structure central to the function of C_13_H_10_N_2_O comprises an α,β-unsaturated ketone moiety, 3-pyridinyl and 4-pyridinyl rings, and a planar conjugated system.	
Shikonin	C_16_H_16_O_5_	238
	The molecular structure central to the function of C_16_H_16_O_5_ comprises a 1,4-naphthoquinone core, a C1-hydroxyl group, and an R-configuration at the C1’ position.	
TEPP-46	C_19_H_20_N_2_O_4_	239
	The molecular structure central to the function of C_19_H_20_N_2_O_4_ comprises 1,3-dicarboxylic acid groups, a central 2-methylpyridine ring, and symmetric aryl ether linkages.	
FX11	C_11_H_8_O_3_	240
	The molecular structure central to the function of C_11_H_8_O_3_ comprises a 1,4-naphthoquinone core, electrophilic β-carbons at the C2 and C3 positions, and the absence of a hydroxyl group at the C1 position.	
Fasentin	C_18_H_18_ClN_3_O_3_	241
	The molecular structure central to the function of C_18_H_18_ClN_3_O_3_ comprises a chlorobenzamide core, a pyridinyl-N-oxide moiety, and a flexible ethoxy linker (─CH_2_CH_2_O─).	
DHEA	C_19_H_28_O_2_	242
	The molecular structure central to the function of C_19_H_28_O_2_ comprises a Δ^5^-androstene skeleton, a C3-ketone group (-C=O), and a C17β-hydroxyl moiety (-OH).	

Note: These drugs exhibit a similar mechanism of action as inhibitors of GLUT proteins. The inhibition of GLUT results in the disruption of cellular glucose uptake, leading to a significant reduction in intracellular NADPH levels. This reduction impairs the reduction of disulfide bonds, causing an accumulation of aberrant disulfide formations. The resulting disulfide stress induces a catastrophic collapse of the actin cytoskeleton, ultimately leading to disulfidptosis. GLUT, glucose transporter; NADPH, reduced nicotinamide adenine dinucleotide phosphate; 3PO, 3-(3-pyridinyl)-1-(4-pyridinyl)-2-propen-1-one; DHEA, dehydroepiandrosterone.

Conversely, current glucose transporter inhibitors often exhibit insufficient target specificity, which not only restricts their anti-tumor effectiveness but may also disrupt the physiological metabolism of normal tissues, such as the brain, erythrocytes, and immune cells, resulting in unacceptable off-target toxicity. For example, while BAY-876 and KL-11743 have shown anti-tumor potential in preclinical studies—with the latter significantly inhibiting NCI–H226 xenograft tumor growth at a dose of 100 mg/kg—there is a lack of clinical data on the safety, tolerability, pharmacokinetic profiles, or definitive efficacy of these agents in humans.^24^ Importantly, research into targets associated with the mechanism of disulfidptosis offers indirect clinical insights for this domain. For instance, an inhibitor of GYS1 is currently undergoing a phase I clinical trial (NCT05249621), which seeks to assess its safety and tolerability in patients with advanced solid tumors and establish the recommended phase II dose.^106^ Although this trial does not directly evaluate glucose transporter inhibitors, as the first drug entering clinical assessment based on a strategy to induce disulfidptosis, its safety data and trial design will provide crucial insights for the subsequent development of a new generation of highly selective glucose transporter inhibitors.

Consequently, future research should prioritize enhancing therapeutic selectivity and overcoming the adaptive resistance mechanisms of cancer cells. Viable strategies include the development of combination therapies or broad-spectrum inhibitors that can simultaneously target multiple key GLUTs. For example, the novel dual-functional inhibitor TH-G313B can concurrently inhibit GLUT1 and GLUT3, thereby reducing metabolic escape through dual blockade.^107^ Additionally, a microcrystalline formulation of BAY-876 is designed to improve tumor-localized drug exposure by optimizing pharmacokinetic properties, potentially reducing systemic toxicity.^108^^,^^109^ Preliminary animal studies indicate that the combined inhibition of GLUT1 and SGLT2 may synergistically suppress tumor growth and extend survival;^110^ however, the specific drug combinations, dosing regimens, and their long-term efficacy and safety necessitate systematic preclinical validation. Another critical strategy involves the development of tumor-targeted drug delivery systems, such as conjugating GLUT inhibitors to tumor-specific antibodies or encapsulating them in nanocarriers, to achieve targeted enrichment of the drug within tumor tissues.^111^

Beyond GLUT antagonism, various pathways converge on disulfidptosis induction. Cystine supply takes place via *de novo* biosynthesis, intracellular proteolysis, or extracellular cystine uptake.^18^^,^^23^ Among these, extracellular cystine import is predominant in tumor cells; increased influx correlates with higher disulfidptosis incidence.^24^ SLC7A11 overexpression, whose gene encodes the cystine importer, critically determines disulfidptosis sensitivity.^24^ A rational therapeutic strategy involves pharmacological upregulation of SLC7A11—via sulforaphane or curcumin—when combined with GLUT blockade to induce glucose deprivation in malignant cells.^112^^,^^113^ Theoretically, this strategy offers high selectivity by simultaneously targeting two prominent metabolic characteristics of cancer cells: elevated cystine uptake and increased reliance on glucose. Preclinical investigations have demonstrated that tumors with high SLC7A11 expression show heightened sensitivity to GLUT inhibitors. Nonetheless, to date, no clinical trials have explicitly implemented a protocol that combines agents such as sulforaphane or curcumin with GLUT1 or SGLT1 inhibitors with the therapeutic aim of inducing disulfidptosis. This observation suggests that the strategy remains at the theoretical and preclinical stages and has not yet been integrated into clinical practice.

In addition to facilitating cystine uptake, the Trx system is critical for cystine reduction. Consisting of various Trxs and thioredoxin reductases (TrxR), this system utilizes NADPH to reduce active-site disulfide bonds in Trx substrates to dithiol. Inhibiting TrxR1 interferes with the reduction of cystine to cysteine, a critical step that may induce disulfidptosis in cells with high SLC7A11 expression.^114^^,^^115^ TrxR inhibitors, such as 2-AAPA and auranofin, trigger disulfidptosis by suppressing TrxR activity.^116^^,^^117^ This inhibition impedes the conversion of cystine to cysteine and significantly depletes NADPH, thereby initiating a cascade of disulfide stress reactions.^115^ A significant limitation of this approach is the inadequate target specificity of the inhibitors, which substantially constrains their clinical applicability.^117^^,^^118^

TrxR is extensively expressed in normal cells, and its active site is susceptible to nonspecific interactions with various drugs, resulting in considerable off-target effects and systemic cytotoxicity.^15^ These risks have been consistently underscored in preclinical studies. For example, in mesothelioma models, auranofin not only demonstrated cytotoxic effects on cancer cells but also exhibited even greater toxicity against normal mesothelial cells.^119^ Although auranofin is approved for the treatment of rheumatoid arthritis and has progressed to phase II clinical trials for certain cancers, including chronic lymphocytic leukemia and lung cancer, there is currently no clinical data supporting its use in patients with malignant mesothelioma.^120^^,^^121^ Another pertinent example is the Trx system inhibitor PX-12, which was the first Trx-1 inhibitor to reach the clinical stage. Its phase I study in patients with advanced solid tumors showed modest disease-stabilizing effects; however, subsequent phase II trials were discontinued due to a complex administration regimen, limited efficacy, and the occurrence of serious adverse effects, such as reversible pneumonitis.^121^^,^^122^ These instances clearly illustrate the narrow therapeutic window associated with the non-selective inhibition of the TrxR/Trx system.

Despite a large body of preclinical studies focused on the development of novel TrxR inhibitors, no candidate drug has yet progressed to phase I or II clinical trials demonstrating unequivocal selectivity for TrxR1 over TrxR2 or other off-targets. Consequently, future advancements in this domain should prioritize two primary objectives: firstly, the initiation of rigorously designed clinical trials for combination therapies targeting both SLC7A11 and GLUT to establish their safety and efficacy in human subjects; secondly, the urgent development of a new generation of inhibitors with high specificity for TrxR1 is essential. These inhibitors should be capable of effectively differentiating TrxR1 from TrxR2 and other selenoproteins, utilizing rational drug design. Achieving these goals is crucial to overcoming the current toxicity limitations and unlocking the translational potential of the disulfidptosis strategy in clinical therapy (Table 4).

**Table 4:** List of SLC7A11 inducers and TrxR inhibitors.

Types	Drugs	Molecular architectural features	Reference
SLC7A11 inducers	Vitamin D	C_27_H_44_O (Vitamin D_3_),	243
		C_28_H_44_O (Vitamin D_2_)	
		The molecular structure central to the function of Vitamin D comprises a secosteroid scaffold, a triene system, a C3β-hydroxyl group, and an aliphatic side chain.	
	Fisetin	C_15_H_10_O_6_	229
		The molecular structure central to the function of C_15_H_10_O_6_ comprises a 3,7-dihydroxychromen-4-one scaffold featuring a catechol moiety (3′,4′-diOH), a C3-hydroxyl group, and distinct protonation sites at C8–H and C6–H.	
	Lactoferrin	UniProt P02788	244
		The molecular structure central to the function of UniProt P02788 comprises iron-binding residues, the lactoferricin (Lfcin) domain, and N-linked glycans.	
	N-acetylcysteine	C_5_H_9_NO_3_S	245
		The molecular structure central to the function of C_5_H_9_NO_3_S comprises a thiol group (-SH), an N-acetyl moiety (-COCH_3_), and a carboxylate group (-COO^-^).	
	GW9662	C_17_H_14_ClN_2_O_2_S	246
		The molecular structure central to the function of C_17_H_14_ClN_2_O_2_S comprises an electrophilic methylene bridge (–CH_2_–), a 2-chlorophenyl group, and a p-nitrobenzylthio moiety (-S–CH_2_–C_6_H_4_–NO_2_).	
	Curcumin	C_21_H_20_O_6_	112
		The molecular structure central to the function of C_21_H_20_O_6_ comprises a symmetric diferuloylmethane scaffold featuring a β-diketone linker, ortho-methoxyphenol groups, and α,β-unsaturated carbonyl moieties.	
	Buthionine-sulfoximine	C_8_H_18_N_2_O_3_S	247
		The molecular structure central to the function of C_8_H_18_N_2_O_3_S comprises a sulfoximine oxime nitrogen moiety (-N-O^-^), a sulfoxide oxygen atom (S=O), and a butyl side chain (-(CH_2_)_3_CH_3_).	
TrxR inhibitors	2-AAPA	C_11_H_13_NO_3_	116
		The molecular structure central to the function of C_11_H_13_NO_3_ comprises an acryloyl group (-CH=CH–C=O), a 4-amino group (-NH_2_), and an acetyl moiety (-COCH_3_).	
	Auranofin	C_20_H_34_AuO_9_PS	117
		The molecular structure central to the function of C_20_H_34_AuO_9_PS comprises a linear Au(I) center, a tetraacetylthioglucose ligand, a triethylphosphine group, and a labile Au–S bond.	
	TRI-1	C_19_H_19_F_3_N_4_O_3_	248
		The molecular structure central to the function of C_19_H_19_F_3_N_4_O_3_ comprises a 4-(trifluoromethyl)phenyl group, an imidazolinone carbonyl/amine moiety, and a 2-fluorophenyl substituent.	

Note: Compounds such as vitamin D, fisetin, lactoferrin, N-acetylcysteine, GW9662, curcumin, and buthionine sulfoximine act as inducers of SLC7A11, leading to its transcriptional up-regulation through a common mechanism. This shared action results in the depletion of NADPH, induction of disulfide stress, and abnormal crosslinking of F-actin, thereby initiating disulfidptosis via cytoskeletal collapse. Similarly, the compounds 2-AAPA, auranofin, and TRI-1 serve as TrxR inhibitors through a convergent mechanism. Inhibition of TrxR disrupts cellular redox homeostasis, causing NADPH depletion and a significant redox imbalance. This disruption leads to abnormal cystine accumulation and subsequent disulfide stress, which facilitates pathological disulfide crosslinking of F-actin filaments. The ensuing cytoskeletal collapse ultimately triggers disulfidptosis. SLC7A11, solute carrier family 7a member 11; NADPH, nicotinamide adenine dinucleotide phosphate (reduced form); TrxR, thioredoxin reductases; 2-AAPA, 2-aminoethyl diphenylborinate.

NADPH serves as a crucial electron donor in organisms, providing the necessary reducing power for anabolic reactions and maintaining REDOX balance. Cancer cells exhibit remarkable metabolic flexibility in maintaining NADPH homeostasis through coordinated activation of multiple biosynthetic routes. The PPP, folate cycle, and substrate-specific catabolism (including lactate, glutamine, and fatty acid oxidation) collectively contribute to REDOX balance, with key enzymatic pathways involving isocitrate dehydrogenase isoforms and malic enzyme playing pivotal regulatory roles.^123^ Inhibiting key enzymes within these pathways can lead to a reduction in NADPH production, thereby triggering disulfidptosis. Oxidative stress inducers, including curcumin, tert-butyl hydroperoxide, berberine, and resveratrol, initiate disulfidptosis by inhibiting the PPP, generating ROS, inhibiting biosynthetic enzymes, and depleting NADPH reserves, thereby disrupting REDOX homeostasis and promoting pathogenic disulfide accumulation.^112^^,^^124^, ^125^, ^126^ While natural products like curcumin and resveratrol have the capacity to induce disulfidptosis through NADPH depletion, their clinical utility is significantly constrained by dose-dependent non-specific cytotoxicity and suboptimal pharmacokinetic properties.^127^ For example, clinical trials involving resveratrol were discontinued due to nephrotoxicity.^128^ Additionally, compounds such as curcumin and berberine encounter obstacles, including rapid metabolism *in vivo* and poor aqueous solubility, leading to inadequate therapeutic exposure.^125^^,^^127^^,^^129^ As a result, the current research emphasis has transitioned from broad-spectrum oxidative stress inducers to the development of highly tumor-selective strategies, such as GLUT inhibitors and SLC7A11 activators. These novel approaches are designed to precisely target the distinct metabolic phenotypes and microenvironmental characteristics of cancer cells, thereby enhancing cytotoxic efficacy while mitigating adverse effects on normal tissues (Table 5).

**Table 5:** List of oxidative stress inducers.

Drugs	Molecular architectural features	Reference
Disulfiram	C_10_H_20_N_2_S_4_	249
	The molecular structure central to the function of C_10_H_20_N_2_S_4_ comprises a disulfide-thiocarbonyl system, symmetric diethylamino side chains, and a bis(thiocarbamate) framework.	
Doxorubicin	C_27_H_29_NO_11_	250
	The molecular structure central to the function of C_27_H_29_NO_11_ comprises a quinone-hydroquinone redox system, a C9 acetyloxy side chain, and a C3’ amino group.	
Vincristine	C_46_H_56_N_4_O_10_	252, 251
	The molecular structure central to the function of C_46_H_56_N_4_O_10_ comprises a C23’ tertiary amine moiety, a C2–C3 π system, a C4 acetoxy group, and a C18’ aldehyde functional group.	
Arsenic trioxide	As_2_O_3_	253
	The molecular structure central to the function of As_2_O_3_ comprises an arsenic(III) (As(III)) center.	
Paclitaxel	C_47_H_51_NO_14_	254
	The molecular structure central to the function of C_47_H_51_NO_14_ comprises a C13 N-acyl-β-phenylisoserine side chain, a C2 benzoyloxy group, an oxetane ring, and acetate groups at the C4, C5, and C20 positions.	
Tert-butyl hydroperoxide	C_4_H_10_O_2_	124
	The molecular structure central to the function of C_4_H_10_O_2_ is functionally defined by a weak O–O bond, a polar O–H bond, and an electrophilic α-carbon.	
Cyclophosphamide	C_7_H_15_Cl_2_N_2_O_2_P	255
	The molecular structure central to the function of C_7_H_15_Cl_2_N_2_O_2_P comprises a bis(2-chloroethyl)amino group, a cyclic phosphoramide ring, and a labile C4–N bond.	
Resveratrol	C_14_H_12_O_3_	126
	The molecular structure central to the function of C_14_H_12_O_3_ comprises an E-configured stilbene scaffold, a 3,5-dihydroxyphenyl group, a 4′-hydroxyl group, and an extended π-conjugation system.	
Curcumin	C_21_H_20_O_6_	112
	The molecular structure central to the function of C_21_H_20_O_6_ comprises a symmetric diferuloylmethane scaffold featuring a β-diketone linker, ortho-methoxyphenol groups, and α,β-unsaturated carbonyl moieties	
Paraquat	C_12_H_14_N_2_^2+^·2Cl^-^	256
	The molecular structure central to the function of C_12_H_14_N_2_^2+^·2Cl^-^ comprises a bipyridinium dication, a planar aromatic system, N-methyl substituents, and a conjugated π-system.	
Melphalan	C_13_H_18_Cl_2_N_2_O_2_	257
	The molecular structure central to the function of C_13_H_18_Cl_2_N_2_O_2_ comprises a bifunctional alkylating bis(2-chloroethyl)amine group, an l-phenylalanine carrier moiety, a chiral α-carbon with L-configuration, and a carboxylic acid functional group.	
Vitamin D	C_27_H_44_O (vitamin D_3_), C_28_H_44_O (vitamin D_2_)	243
	The molecular structure central to the function of vitamin D comprises a secosteroid scaffold, a triene system, a C3β-hydroxyl group, and an aliphatic side chain.	
Pterostilbene	C_16_H_16_O_3_	258
	The molecular structure central to the function of C_16_H_16_O_3_ comprises 3,5-dimethoxy groups, a 4′-hydroxyl group, and a trans-configured double bond.	
Berberine	C_20_H_18_NO_4_^+^	125
	The molecular structure central to the function of C_20_H_18_NO_4_^+^ comprises a quaternary ammonium group (N^+^), a methylenedioxy bridge (-O–CH_2_–O-), and methoxy substituents (-OCH_3_).	
TRI-1	C_19_H_19_F_3_N_4_O_3_	248
	The molecular structure central to the function of C_19_H_19_F_3_N_4_O_3_ comprises a 4-(trifluoromethyl)phenyl group, an imidazolinone carbonyl/amine moiety, and a 2-fluorophenyl substituent.	
2DG	C_6_H_12_O_5_	259
	The molecular structure central to the function of C_6_H_12_O_5_ comprises a C2-hydrogen atom and a C1-hydroxyl group.	
Cytochalasin B	C_29_H_37_NO_5_	260
	The molecular structure central to the function of C_29_H_37_NO_5_ comprises a macrolide ring with indole fusion, an epoxide group at C7─C8–O, and a benzylidene moiety (-CH=C_6_H_5_).	
DHEA	C_19_H_28_O_2_	242
	The molecular structure central to the function of C_19_H_28_O_2_ comprises a Δ^5^-androstene skeleton, a C3-ketone group (-C=O), and a C17β-hydroxyl moiety (-OH).	
6-Aminonicotinamide	C_6_H_7_N_3_O	261
	The molecular structure central to the function of C_6_H_7_N_3_O comprises a C6-amino group (-NH_2_), a pyridine ring nitrogen atom, and a C3-carboxamide moiety (-CONH_2_).	
AG-120	C_28_H_28_F_3_N_7_O_3_	262
	The molecular structure central to the function of C_28_H_28_F_3_N_7_O_3_ comprises a quinolin-2(1H)-one core, a 6-(trifluoromethyl)benzyl group, a 2,4-diaminopyrimidine moiety, and an aminoethyl linker (–CH_2_CH_2_NH–).	

Note: The compounds disulfiram, doxorubicin, vincristine, arsenic trioxide, paclitaxel, tert-butyl hydroperoxide, cyclophosphamide, resveratrol, curcumin, paraquat, melphalan, vitamin D, pterostilbene, berberine, TRI-1, 2DG, cytochalasin B, DHEA, 6-aminonicotinamide, and AG-120 collectively act as inducers of oxidative stress through a unified mechanism. These agents induce the depletion of NADPH, which leads to the accumulation of cystine and subsequent disulfide stress. This process ultimately results in cytoskeletal collapse and culminates in disulfidptosis. NADPH, reduced nicotinamide adenine dinucleotide phosphate; 2DG, 2-deoxy-d-glucose; DHEA, dehydroepiandrosterone.

The Rac1–WRC signaling axis plays a crucial role in the regulation of disulfidptosis by promoting actin branch nucleation.^55^ Although activation of this pathway holds promise as an anti-cancer strategy, existing Rac1 inhibitors, such as NSC23766, EHT1864, and EHop-016, are hindered by low potency and the necessity for high doses.^130^^,^^131^ Specifically, NSC23766 has an IC50 of 50 μM, which is significantly higher than concentrations that are clinically viable, thereby limiting its potential for clinical application despite its ability to enhance chemosensitivity in non-small cell lung cancer.^132^ Additionally, issues with specificity remain problematic: EHop-016 inhibits CDC42 at concentrations greater than 5 μM, thus affecting therapeutic selectivity,^130^ while EHT1864 indiscriminately targets Rac1 isoforms (Rac1b/Rac2/Rac3) and related paralogs, potentially leading to off-target physiological effects.^133^ In comparison to the *de novo* development of novel inhibitors with high molecular specificity, a more practical approach involves exploiting the distinct physicochemical and biological characteristics of the tumor microenvironment to enhance the tumor-targeting efficacy of existing pharmaceuticals through advanced drug delivery systems. For example, nanocarrier systems designed for passive or active targeting can be employed to augment drug accumulation within tumor tissues.^134^ Utilizing the enhanced permeability and retention effect observed in solid tumors, the encapsulation of a Rac1 inhibitor within nanoparticles can substantially elevate its localized concentration at the tumor site. This strategy permits a reduction in systemic dosage while improving the drug's “functional selectivity” and minimizing exposure risks to normal tissues.^135^ Additionally, by functionalizing the surface of nanocarriers with ligands, such as antibodies or aptamers, that specifically bind to tumor cell surface antigens (*e.g.*, folate receptor, transferrin receptor), active targeted delivery can be achieved, thereby further improving drug localization and accumulation efficiency of the drug within tumor cells.^136^

From a clinical perspective, research on disulfidptosis demonstrates promising therapeutic potential. Researchers are investigating the potential of leveraging the mechanism of disulfidptosis to develop novel anti-tumor therapeutics, including the recently identified peroxisome proliferator-activated receptor γ (PPARγ) antagonists in oral squamous cell carcinoma. Inhibition of PPARγ can suppress tumor progression by inducing ferroptosis through the up-regulation of heme oxygenase 1 (HMOX1) and disulfidptosis via the up-regulation of SLC7A11. Additionally, PPARγ inhibition enhances the tumor microenvironment by increasing the presence of conventional dendritic cells and CD8^+^ T cells, thereby augmenting the immune response against tumors.^137^ However, although such strategies have shown promise in preclinical studies, no phase I/II clinical trials specifically designed to evaluate the efficacy and safety of PPARγ antagonists in patients with solid tumors have been registered or had their study protocols disclosed to date. Moreover, the mechanism by which PPARγ antagonists induce disulfidptosis remains incompletely elucidated, and only limited mechanistic investigations have been conducted so far. Moreover, unresolved pharmacological challenges, such as inadequate bioavailability and insufficient tumor-targeting specificity, pose significant obstacles to the therapeutic translation of these compounds. Building on the current bioinformatics insights into disulfidptosis, future research should aim to elucidate a more defined pathway linking drug development and disulfidptosis. This would facilitate advancements in disulfidptosis research and its subsequent clinical application (as shown in Fig. 5). Glutaminase (GLS) inhibitors, such as CB-839, induce disulfidptosis by inhibiting GSH synthesis, thereby disrupting cellular REDOX homeostasis and promoting pathological disulfide accumulation.^138^ However, GLS inhibitors similarly exhibit the aforementioned issues (Table 6).

**Figure 5:**
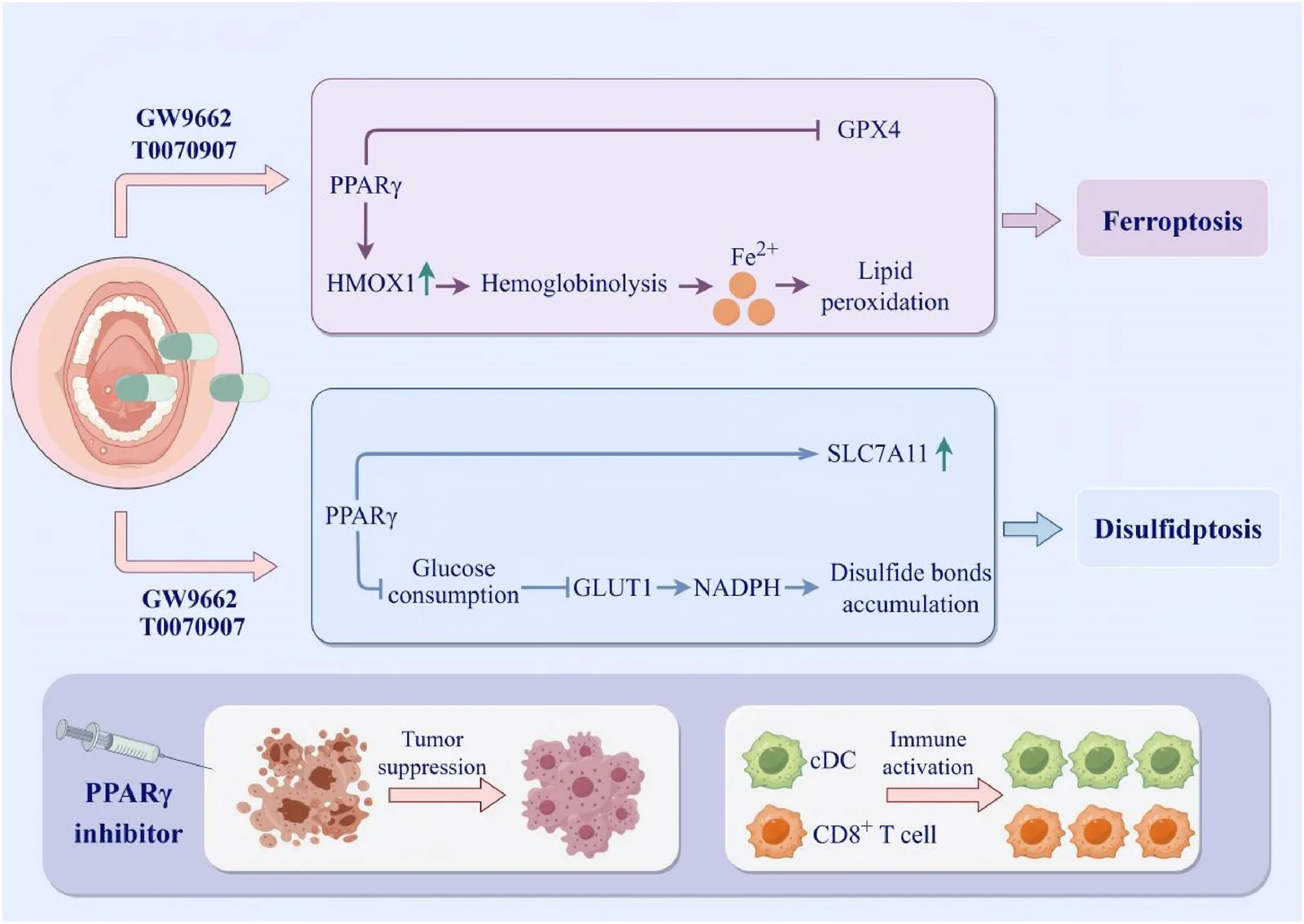
Efficacy and mechanisms of inducing disulfidptosis and ferroptosis through PPARγ inhibitors for the treatment of OSCC. The application of PPARγ inhibitors, such as GW9662 and T0070907, effectively suppressed PPARγ activity and resulted in a significant reduction in tumor volume within an orthotopic xenograft mouse model of OSCC. Furthermore, these inhibitors modulated the immune microenvironment and activated immune cells, leading to a marked increase in the number of cDCs and CD8^+^ T cells within tumor tissues. This enhancement of the anti-tumor immune response was mechanistically linked to the up-regulation of the HMOX1 gene, accompanied by increased expression of its transcription factor p-NRF2 and decreased expression of GPX4. The up-regulation of HMOX1 facilitated hemoglobin degradation, elevated intracellular iron levels, induced lipid peroxidation, and ultimately resulted in ferroptosis. Additionally, PPARγ inhibition led to the up-regulation of SLC7A11 gene expression. Concurrently, PPARγ inhibition was associated with reduced cellular glucose consumption, diminished GLUT1 expression, and decreased intracellular NADPH levels. The overexpression of SLC7A11, in the context of insufficient NADPH supply, promoted the abnormal accumulation of disulfide bonds in cancer cells, culminating in disulfidptosis. The figure was created by Figdraw. OSCC, oral squamous cell carcinoma; PPARγ, peroxisome proliferator-activated receptor gamma; cDCs, conventional dendritic cells; GLUT1, glucose transporter 1; NADPH, nicotinamide adenine dinucleotide phosphate (reduced form); SLC7A11, solute carrier family 7a member 11; GPX4, glutathione peroxidase 4; HMOX1, heme oxygenase 1.

**Table 6:** List of WRC-Rac1 inhibitors, PPARγ inhibitors, and GR inhibitors.

Types	Drugs	Molecular architectural features	Reference
WRC-Rac1 inducers	NSC23766	C_22_H_23_N_7_·3HCl	139
		The molecular structure central to the function of C_22_H_23_N_7_·3HCl comprises a 2-aminopyrimidine core, a catechol moiety, and an N-methylindazole group.	
	EHT1864	C_24_H_26_ClN_5_O_2_	131
		The molecular structure central to the function of C_24_H_26_ClN_5_O_2_ comprises a quinazolinone carbonyl/amine moiety, a protonated tertiary amine group (-N^+^H(CH_3_)_2_), and a 4-chlorophenyl substituent.	
	Ehop-016	C_26_H_26_N_2_O_4_S	130
		The molecular structure central to the function of C_26_H_26_N_2_O_4_S comprises a 2-naphthyl group, a thiazolidinedione core, and a carboxylate tail (-COO^-^).	
PPARγ inhibitors	GW9662	C_17_H_14_ClN_2_O_2_S	246
		The molecular structure central to the function of C_17_H_14_ClN_2_O_2_S comprises an electrophilic methylene bridge (–CH_2_–), a 2-chlorophenyl group, and a p-nitrobenzylthio moiety (-S–CH_2_–C_6_H_4_–NO_2_).	
	T0070907	C_17_H_13_ClN_2_O_2_S	263
		The molecular structure central to the function of C_17_H_13_ClN_2_O_2_S comprises an electrophilic methylene bridge (–CH_2_–), a 2-chlorophenyl group, and a p-nitrobenzylthio moiety (-S–CH_2_–C_6_H_4_–NO_2_).	
GR inhibitors	Buthionine-sulfoximine	C_8_H_18_N_2_O_3_S	247
		The molecular structure central to the function of C_8_H_18_N_2_O_3_S comprises a sulfoximine oxime nitrogen moiety (-N-O^-^), a sulfoxide oxygen atom (S=O), and a butyl side chain (-(CH_2_)_3_CH_3_).	
	2-AAPA	C_11_H_13_NO_3_	116
		The molecular structure central to the function of C_11_H_13_NO_3_ comprises an acryloyl group (-CH=CH–C=O), a 4-amino group (-NH_2_), and an acetyl moiety (-COCH_3_).	
	CB-839	C_14_H_13_F_6_N_3_O_3_S	264
		The molecular structure central to the function of C_14_H_13_F_6_N_3_O_3_S comprises a bis(trifluoromethyl)benzamide core, a sulfonamide linker (─SO_2_NH─), and a 1,3,4-thiadiazole ring	
	Auranofin	C_20_H_34_AuO_9_PS	117
		The molecular structure central to the function of C_20_H_34_AuO_9_PS comprises a linear Au(I) center, a tetraacetylthioglucose ligand, a triethylphosphine group, and a labile Au–S bond.	

Note: NSC23766, EHT1864, and Ehop-016 act as WRC-Rac1 inhibitors through a convergent mechanism. Their inhibition of Rac1 disrupts actin dynamics, promotes aberrant F-actin disulfide crosslinking, and induces cytoskeletal collapse, ultimately culminating in disulfidptosis. Similarly, GW9662 and T0070907 serve as PPARγ inhibitors via a convergent mechanism. Their covalent inhibition of PPARγ leads to NADPH depletion, which results in F-actin disulfide crosslinking and ultimately induces disulfidptosis. Furthermore, buthionine sulfoximine, 2-AAPA, CB-839, and auranofin function as GR inhibitors through a convergent mechanism. Their inhibition of GR results in a loss of NADPH reduction capacity, leading to the accumulation of GSSG and thiol stress. This causes disulfide overload, promotes F-actin disulfide crosslinking, and induces cytoskeletal collapse, ultimately triggering disulfidptosis. GR, glutathione reductase; Rac1, Ras-related c3 botulinum toxin substrate 1; WRC, WAVE regulatory complex; NADPH, reduced nicotinamide adenine dinucleotide phosphate; GSSG, glutathione disulfide; 2-AAPA, 2-aminoethyl diphenylborinate.

Currently, anti-tumor strategies designed to induce disulfidptosis, while demonstrating considerable potential, universally encounter significant limitations, presenting a complex landscape of concurrent opportunities and challenges. Among these strategies, the most direct approach involves targeting to induce disulfidptosis by inhibiting glucose uptake in cancer cells. Small-molecule GLUT inhibitors, such as BAY-876 and KL-11743, have demonstrated clear anti-tumor activity across various preclinical tumor models, thereby validating the feasibility of this pathway.^100^, ^101^, ^102^ However, their clinical translation is impeded by two primary challenges: first, cancer cells exhibit remarkable metabolic plasticity, whereby inhibition of major GLUT subtypes (*e.g.*, GLUT1) can lead to metabolic escape through the up-regulation of other GLUT subtypes or the engagement of alternative transporters (*e.g.*, SGLT1), thus compromising the durability of monotherapy efficacy.^103^^,^^104^ Second, existing GLUT inhibitors generally lack subtype selectivity, potentially resulting in the broad inhibition of essential glucose uptake in normal tissues such as the brain and heart, thereby introducing significant systemic toxicity risks that represent a critical vulnerability in clinical application.^24^

Targeting downstream nodes of the disulfidptosis execution pathway constitutes a notable class of strategies, including the inhibition of TrxR and intervention in NADPH generation pathways. TrxR inhibitors, such as auranofin and 2-AAPA, alongside natural compounds like curcumin and resveratrol, have been demonstrated to induce disulfidptosis in experimental systems.^115^^,^^127^ However, these agents frequently exhibit low target specificity and significant off-target effects, resulting in a limited therapeutic window.^127^ A pertinent example is the Trx inhibitor PX-12, whose clinical development was discontinued due to severe adverse reactions, highlighting a cautionary precedent for similar strategies. Furthermore, most natural products encounter pharmaceutical challenges, such as low bioavailability and poor chemical stability.^121^^,^^122^ Intervention targeting the upstream Rac1–WRC signaling axis is also under investigation; however, existing inhibitors like NSC23766 are limited by insufficient potency and inadequate selectivity, which impede their potential as drug candidates.^139^ Future advancements are likely to exploit nano-targeted delivery systems to improve drug accumulation within tumor tissues, effectively compensating for their limited molecular selectivity. Additionally, novel strategies, including the application of PPARγ antagonists, have preliminarily revealed connections between disulfidptosis and the tumor immune microenvironment, thereby offering a theoretical basis for combination immunotherapy.^137^ However, the exact molecular mechanisms through which they induce disulfidptosis remain inadequately understood. These approaches also face common challenges, such as low bioavailability and a lack of robust clinical efficacy evidence.

In conclusion, the primary challenge facing current therapeutic strategies based on disulfidptosis is achieving a balance between potent cytotoxicity and high selectivity to ensure effective and safe clinical translation. Future advancements are unlikely to arise from singular approaches; rather, they will require multidisciplinary, integrated systemic strategies. These strategies should encompass rational drug design informed by structural biology and artificial intelligence to develop highly selective inhibitors targeting key proteins such as TrxR1. Additionally, the formulation of intelligent combination therapies that can anticipate and counteract metabolic compensation is essential. Furthermore, the application of targeted delivery technologies, including antibody-drug conjugates and nanocarriers, is crucial for precise drug accumulation at tumor sites. Only through such comprehensive innovation can the significant scientific discovery of disulfidptosis be successfully translated into clinical outcomes that benefit cancer patients.

### Combined therapy strategies based on disulfidptosis

Immune checkpoint inhibitors (ICIs) have fundamentally transformed the therapeutic landscape for various malignancies by reactivating T cell-mediated anti-tumor responses through the inhibition of critical negative regulatory pathways, including cytotoxic T-lymphocyte-associated protein 4 (CTLA-4) and programmed death-1 (PD-1)/programmed death-ligand 1 (PD-L1).^140^ Nevertheless, their clinical effectiveness is often limited by primary or acquired resistance in a significant subset of patients, further exacerbated by intertumoral heterogeneity.^141^ To address these challenges, researchers are actively investigating combinatorial approaches that integrate ICIs with the induction of disulfidptosis.

The pharmacological targeting of SLC7A11 or its associated pathways under conditions of glucose deprivation theoretically induces disulfidptosis, which in turn releases tumor-associated antigens and remodels the tumor immune microenvironment (TIME) to facilitate immunogenic cell death (ICD).^142^ This process potentially “primes” the tumor for an enhanced response to ICIs. Although there is a compelling theoretical basis and correlative evidence suggesting a link between disulfidptosis biomarker signatures and immune checkpoint expression in cancers such as thyroid carcinoma,^143^ no peer-reviewed *in vivo* studies have yet provided direct evidence of synergistic tumor regression in immunocompetent murine models, particularly those with high SLC7A11 expression such as lung adenocarcinoma, through the combined inhibition of SLC7A11 (either pharmacologically or genetically) and PD-1/PD-L1 blockade.^142^^,^^144^^,^^145^ This significant lack of empirical evidence restricts the strategy to a promising but as yet unvalidated hypothesis.

In a broader context, the integration of ICIs with agents aimed at metabolic reprogramming to induce disulfidptosis has given rise to numerous therapeutic concepts. However, the clinical translation of these concepts remains challenging. The approach targeting glutamine metabolism serves as a case in point: the GLS inhibitor CB-839 showed considerable promise in preclinical studies by enhancing CD8^+^ T cell infiltration and function, as well as up-regulating interferon-gamma (IFN-γ)-associated inflammatory signatures in colon cancer and melanoma models when used in conjunction with anti-PD-1/PD-L1 antibodies.^146^^,^^147^ Despite this preclinical success, the outcomes in human trials were disappointing. A phase II trial (NCT04265534) in non-small cell lung cancer was prematurely terminated due to a lack of sufficient clinical benefit, while another phase II study (NCT02771626) in PD-1-refractory melanoma reported only “modest” objective response rates without providing detailed objective response rate or progression-free survival data.^148^ These setbacks highlight the significant translational gap between animal models and human patients. Similarly, other metabolic targeting strategies, such as GLUT inhibitors, PPP inhibitors, and Rac1/PAK1 inhibitors,^149^^,^^150^ currently lack clinical trial data to substantiate their synergistic effects with ICIs^151^ (as shown in Fig. 6).

**Figure 6:**
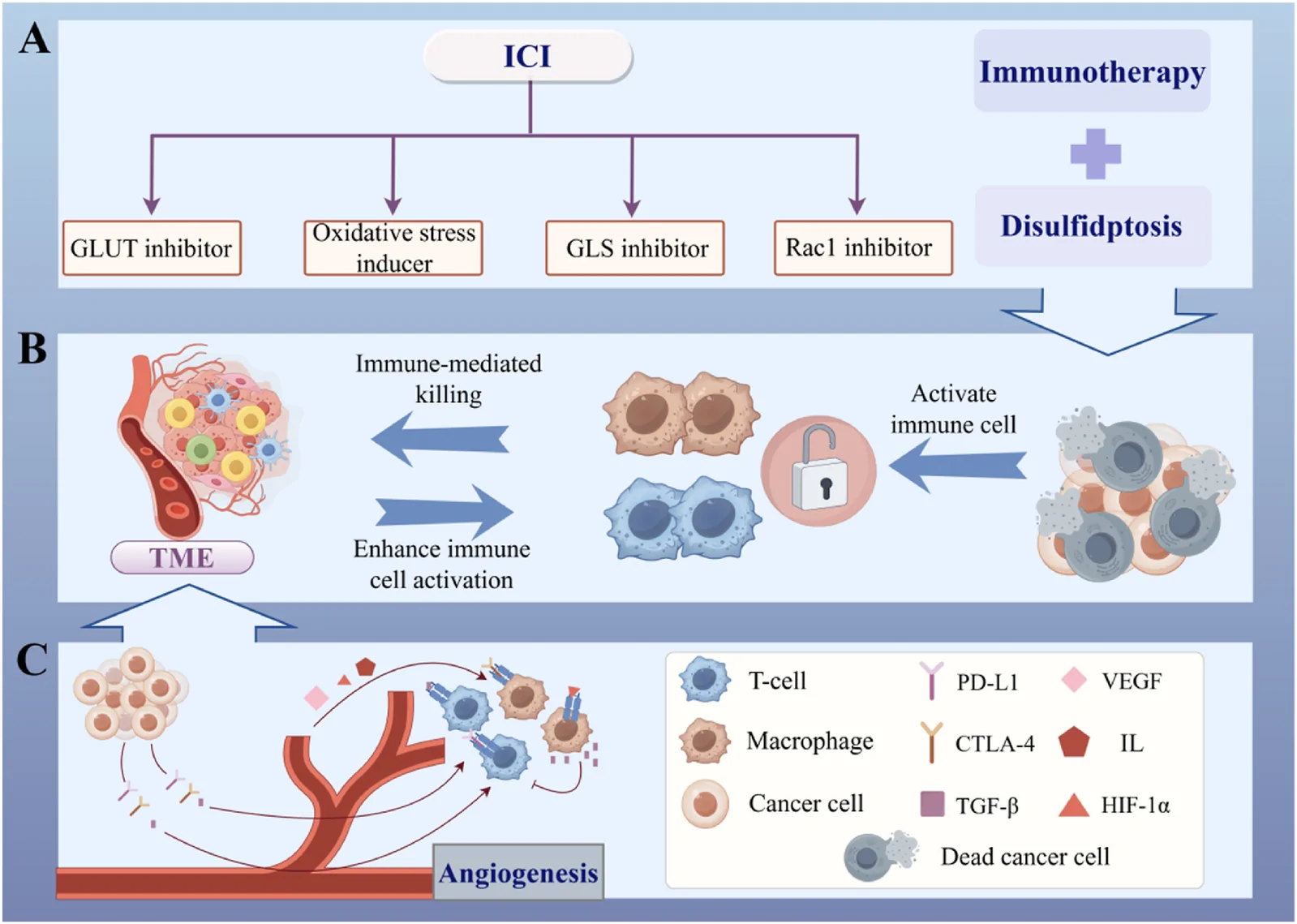
Combined strategies of disulfidptosis and immunotherapy. **(A)** Mechanism of combination therapy: ICIs exhibit synergistic effects when combined with agents that induce disulfidptosis, such as GLUT inhibitors, oxidative stress inducers, GLS inhibitors, and Rac1 suppressors. This synergy enhances therapeutic efficacy by promoting coordinated metabolic cell death and immune activation. **(B)** Tumor immune evasion: Tumor cells evade immune surveillance by secreting immunosuppressive factors, including PD-L1, CTLA-4, and TGF-β, which inhibit T-cell activity. The hypoxic TME, characterized by the stabilization of HIF-1α, overexpression of VEGF, and dysregulation of interleukins, facilitates angiogenesis and tumor progression. Simultaneously, tumor-derived signals promote the polarization of macrophages towards an immunosuppressive phenotype, thereby reinforcing TME-mediated immunosuppression. **(C)** Therapeutic synergy: Agents targeting disulfidptosis directly suppress or eliminate tumor cells, thereby reducing their immunosuppressive capacity. This effect, in conjunction with ICI-mediated checkpoint blockade, enhances T-cell activation, leading to effective tumor eradication and remodeling of the TME. This process thus establishes a self-amplifying therapeutic loop. The figure was created by Figdraw. ICIs, immune checkpoint inhibitors; GLUT, glucose transporter; GLS, glutaminase; Rac1, ras-related c3 botulinum toxin substrate 1; PD-L1, programmed death-ligand 1; CTLA-4, cytotoxic T-lymphocyte-associated protein 4; TGF-β, transforming growth factor-beta; TME, tumor microenvironment; HIF-1α, hypoxia-inducible factor 1-alpha; VEGF, vascular endothelial growth factor.

Moreover, combinatorial therapies encounter substantial challenges, including elevated toxicity and intricate resistance mechanisms. ICIs are associated with immune-related adverse events, with both incidence and severity heightened in combination therapies, such as those involving PD-1 and CTLA-4 inhibitors.^152^, ^153^, ^154^ These adverse events can lead to severe complications and necessitate the discontinuation of treatment.^155^ A critical issue is the lack of biomarkers that can accurately identify patients who are likely to benefit from specific “metabolism-immunotherapy” combinations, thereby complicating the design of clinical trials and the selection of suitable patients.^156^^,^^157^ Although strategies such as disulfidptosis induction and targeting metabolic nodes present innovative methods to overcome ICI resistance, the transition from promising preclinical findings to validated clinical evidence is a lengthy and challenging process.

Recent findings also suggest that integrating disulfidptosis with nanomaterials may serve as an effective approach for cancer therapy. Numerous combinatorial therapeutic strategies targeting disulfidptosis have been developed, including photodynamic therapy, nanomediated chemotherapy, glucose deprivation-oxidative stress co-targeting, photothermal-glucose deprivation synergy, endoplasmic reticulum (ER) stress-glucose transport co-inhibition, enzyme-mediated starvation therapy, targeted prodrug delivery, ultrasound-guided photothermal therapy, and combinatorial delivery systems.

One therapeutic strategy involves harnessing the direct cytotoxic effects of nanomaterials to induce cancer cell death.^158^ The alternative function is to serve as a nanocarrier for drug delivery, aiming to enhance the sensitivity of chemotherapy or radiotherapy, or to modulate intracellular signaling and homeostasis to induce cancer cell death. This mechanism was initially observed in studies investigating disulfidptosis in cancer treatment. For instance, the innovative nanoliposome SC@Lip, which integrates sorafenib and the acoustic sensitizer Chlorophyll e6, promotes disulfidptosis in cancer cells via lipid peroxidation, thereby augmenting the anti-cancer efficacy of sonodynamic therapy.^159^ Additionally, a novel nano-metal–organic framework, Th–Ir-DBB/Dig, addresses the limitations of radiotherapy by targeting mitochondria and releasing digoxin (digitonin), resulting in potent anti-tumor effects under low-dose X-ray irradiation. Th–Ir-DBB/Dig is designed to release digoxin in the acidic TME, triggering cholesterol and glucose depletion, reduced GSH levels, induction of cancer cell disulfidptosis, and increased sensitivity to RT-RDT^160^ (as shown in Fig. 7).

**Figure 7:**
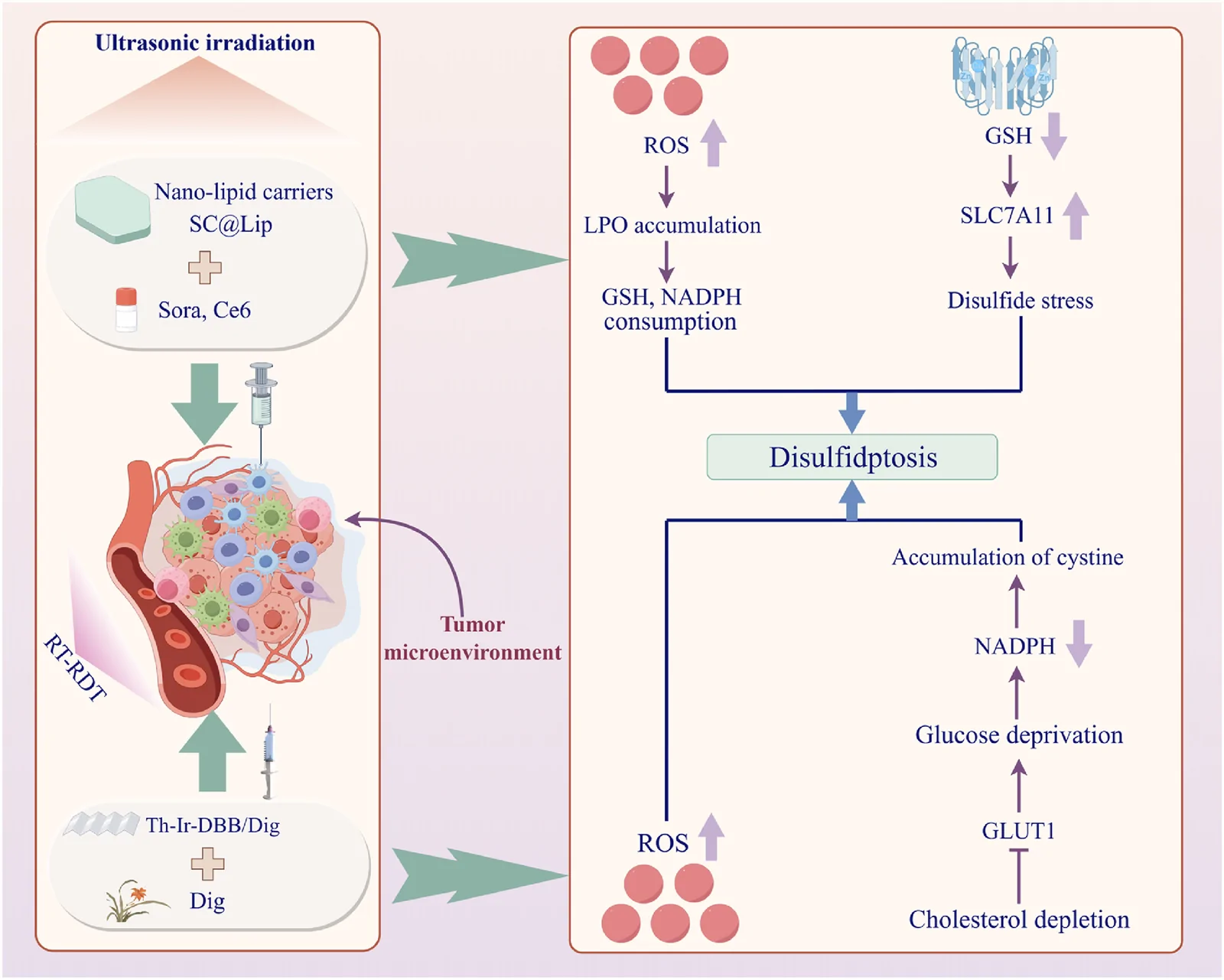
Novel nanomaterials combined with disulfidptosis for cancer treatment strategies. The nanoliposomes SC@Lip, encapsulating Sora and the photosensitizer Ce6, exert potent anti-tumor activity within the TME. Upon exposure to ultrasound irradiation, SC@Lip generates abundant ROS, which subsequently induce extensive intracellular lipid peroxidation, culminating in the accumulation of LPO. Concurrently, SC@Lip depletes intracellular GSH levels, markedly up-regulates the expression of the SLC7A11 protein, and induces disulfide bond stress. Furthermore, the pronounced depletion of intracellular reducing agents, such as GSH and NADPH, impairs the cell's ability to transport cystine, ultimately precipitating disulfidptosis. The mitochondrial-targeted nano-metal–organic framework Th–Ir-DBB/Dig consists of a Th_6_O_4_(OH)_4_ secondary building unit and an Ir(DBB) (ppy)^2+^ photosensitive ligand, which is loaded with Dig. Within the acidic TME, Th–Ir-DBB/Dig releases Dig, resulting in the down-regulation of GLUT1 expression through cholesterol depletion and a consequent reduction in glucose uptake by cancer cells. The diminished glucose consumption leads to decreased NADPH levels, causing intracellular cystine accumulation. Concurrently, RT-RDT elevates ROS levels within the TME, thereby increasing the sensitivity of cancer cells to ROS and ultimately inducing disulfidptosis. The figure was created by Figdraw. ROS, reactive oxygen species; Dig, digitonin; Sora, sorafenib; LPO, lipid peroxides; GSH, glutathione; SLC7A11, solute carrier family 7a member 11; NADPH, nicotinamide adenine dinucleotide phosphate (reduced form); GLUT1, glucose transporter 1; RT-RDT, radiotherapy-photodynamic therapy; TME, tumor microenvironment.

The nanomediated chemotherapeutic agent GCI@nanofilm employs a GOx/CAT enzymatic cascade to induce glucose deprivation and oxygen generation, thereby triggering disulfidptosis while alleviating hypoxia and ROS to enhance photodynamic efficacy.^161^ CGBH NNs target glucose deprivation and oxidative stress via their inherent components: glucose oxidase (GOx) depletes glucose and NADPH, buthionine sulfoximine (BSO) inhibits GSH synthesis, and CGBH NNs generate ROS, collectively inducing disulfidptosis in tumors with high SLC7A11 expression.^162^ The Pd_2_Sn@GOx-SP nanorods integrate photothermal and glucose deprivation strategies. The GOx-induced glucose starvation results in NADPH depletion and actin disulfide stress, leading to disulfidptosis. This process is enhanced by photothermally augmented, enzyme-catalyzed ROS surges, which induce pyroptosis-mediated ICD.^163^ The combination therapy of G157 with BAY-876/KL-11743 targets both ER stress and glucose transport mechanisms. The inhibition of glucose uptake and GLUTs activity reduces NADPH levels, promoting disulfide accumulation and disulfidptosis. Simultaneous ER stress mitigation eliminates cytoprotective responses, increasing cellular susceptibility to death.^164^ GOx@FBSP metal–organic frameworks (MOFs) utilize enzyme-driven starvation. Glucose deprivation initiates SLC7A11-dependent disulfidptosis while simultaneously activating ferroptosis, pyroptosis, and ICD.^165^^,^^166^ The Pro@FLNC system is a targeted prodrug delivery mechanism. The GSH-responsive release of Pro@FLNC depletes intra-tumoral GSH and NADPH, triggering SLC7A11-dependent disulfidptosis. Photodynamic therapy-induced ROS reconfigures the immunogenic TME, effectively synergizing to exploit metabolic vulnerabilities.^167^ ICG@C_3_F_8_-KL nanobubbles facilitate ultrasound-guided photothermal therapy. Inhibition of GLUTs induces glucose starvation, which triggers SLC7A11-dependent disulfidptosis, leading to the release of tumor antigens. This process activates dendritic cells and CD8^+^ T cells, thereby enhancing the efficacy of ICIs through a metabolic-immunological synergy.^168^ The SPCP/CCP@Bay system employs a combinatorial delivery strategy. Ultrasound-triggered release of Bay-876 and cysteine results in GLUT1 inhibition and NADPH depletion, causing F-actin collapse and disulfidptosis. Simultaneously, ROS generation induces ICD, which remodels the TME to promote DC maturation, CD8^+^ T cell and natural killer cell infiltration, and downregulation of PD-L1. This therapeutic regimen synergizes with anti-PD-1 therapy to elicit systemic anti-tumor immunity, thereby suppressing metastasis and recurrence^111^ (Table 7).

**Table 7:** List of combined therapies.

Formulations	Therapy types	Mechanisms	Reference
Th–Ir–DBB/Dig	Photodynamic therapy	The therapeutic efficacy of Th–Ir–DBB/Dig is driven by acid-triggered digitonin release in the tumor microenvironment, inducing disulfidptosis to enhance RT-RDT and reduce immune checkpoints via cholesterol depletion.	160
GCI@nanofilm	Nanomediated chemotherapy	GCI@nanofilm works through a GOx/CAT enzymatic cascade causing glucose starvation and oxygen production, triggering disulfidptosis and reducing hypoxia and ROS to boost photodynamic therapy.	161
CGBH NNs	Starvation-oxidative stress combined therapy	CGBH NNs utilize GOx-induced glucose and NADPH depletion, along with BSO-mediated GSH inhibition, to generate ROS and induce disulfidptosis in SLC7A11-high tumors.	162
Pd_2_Sn@GOx-SP nanorods	Photothermal-starvation combined therapy	Pd_2_Sn@GOx-SP nanorods cause glucose deprivation and NADPH depletion, leading to actin disulfide stress and disulfidptosis, while enhancing ROS storms and triggering pyroptosis-mediated immunogenic cell death.	163
G157 plus BAY-876, KL-11743	Combined inhibition of ER stress and GLUT	The therapeutic efficacy of G157 with BAY-876 and KL-11743 is achieved by glucose deprivation and GLUT inhibition, leading to NADPH depletion, disulfide accumulation, and disulfidptosis, with initial ER stress offering cellular protection but its removal increasing cell death sensitivity.	164
GOx@FBSP MOFs	Enzyme-mediated starvation therapy	GOx@FBSP MOFs work through glucose deprivation-induced SLC7A11-dependent disulfidptosis, activating ferroptosis and pyroptosis, and triggering immunogenic cell death.	165
Pro@FLNC	Targeted prodrug delivery	Pro@FLNC uses GSH-responsive delivery to deplete intra-tumoral GSH/NADPH, inducing SLC7A11-dependent disulfidptosis, while also enabling photodynamic therapy to generate ROS for tumor microenvironment remodeling and targeting metabolic vulnerabilities for enhanced anticancer effects.	167
ICG@C3F8-KL NBs	Ultrasound-guided photothermal therapy	The therapeutic efficacy of ICG@C3F8-KL NBs is achieved by inhibiting GLUT, causing glucose deprivation and SLC7A11-dependent disulfidptosis, which releases tumor antigens to activate dendritic and CD8^+^ T cells, enhancing immune checkpoint inhibition.	168
SPCP/CCP@Bay	Combined delivery therapy	SPCP/CCP@Bay works by ultrasound-triggered release of Bay-876 and cysteine, leading to GLUT1 inhibition, NADPH depletion, F-actin contraction, and disulfidptosis, along with ROS generation for immunogenic cell death. This remodels the tumor microenvironment to boost dendritic cell maturation, CD8^+^ T/NK cell infiltration, and PD-L1 down-regulation, synergizing with anti-PD-1 therapy to strengthen systemic antitumor immunity and prevent metastasis and recurrence.	111

Note: RT-RDT, radiation therapy-radiodynamic therapy; GOx, glucose oxidase; CAT, catalase; BSO, buthionine sulfoximine; GSH, glutathione; SP, photosensitizer; ER, endoplasmic reticulum; GLUT, glucose transporter; MOFs, metal–organic frameworks; Pro, prodrug; ICG, indocyanine green; NBs, nanobubbles; DC, dendritic cell; SPCP, sonodynamic-pseudo-conjugate-polymer; CCP, cystine-containing polymer; NADPH, reduced nicotinamide adenine dinucleotide phosphate; F-actin, filamentous actin; NK, natural killer cell; PD-L1, programmed death-ligand 1; Anti-PD-1, anti-programmed death-1 antibody.

While combination therapeutic strategies based on nanomaterial-induced disulfidptosis demonstrate significant potential in preclinical research, their clinical translation faces substantial challenges and remains in the nascent exploratory phase. As of now, no clinical trials specifically targeting “nanomaterial-based combination therapy for disulfidptosis” have been registered or initiated, indicating that this domain is still firmly within the preclinical research stage.^169^^,^^170^ The primary bottlenecks include: firstly, a critical deficiency in data regarding biocompatibility and long-term toxicity. The long-term accumulation behavior and metabolic pathways of nanosystems containing complex components, such as heavy metals, enzymes, or photosensitizers, *in vivo*, as well as their potential off-target toxicity to normal tissues with active glucose metabolism (*e.g.*, brain tissue and immune cells), have not been systematically evaluated.^171^^,^^172^ Secondly, the pharmacokinetic profiles of nanoformulations are exceedingly complex and challenging to predict. The biodistribution of these agents in humans, their efficacy in traversing biological barriers, their responsiveness within the highly heterogeneous TME (such as sensitivity to pH and GSH concentration), and the controllability of drug release are critical scientific challenges that need to be addressed during the transition from animal models to clinical applications.^171^^,^^172^ Additionally, as disulfidptosis has been recently identified as a mode of cell death, its complete regulatory network, potential resistance mechanisms, and heterogeneity across various tumor types remain insufficiently characterized. This lack of understanding introduces significant uncertainties for forthcoming clinical trials, particularly concerning patient stratification, biomarker selection, and endpoint definition.^173^

Despite these challenges, the integration of disulfidptosis mechanisms with multifunctional nanotechnology offers a novel research paradigm and strategic framework for cancer therapy. To facilitate clinical translation in this domain, future research should prioritize the following areas: Firstly, there is a pressing need for a systematic exploration and optimization of combination therapeutic strategies. This involves the precise regulation of the composition, dosing, timing, and application window of nanosystems to achieve optimal synergistic effects with existing modalities such as chemotherapy, radiotherapy, and immunotherapy, to enhance efficacy while rigorously controlling adverse effects. Secondly, efforts must be intensified to deepen the understanding of the molecular mechanisms underlying disulfidptosis. A comprehensive elucidation of disulfidptosis-related upstream and downstream regulatory networks, key triggering conditions, and interactions with other cellular signaling pathways will provide a robust theoretical foundation for the development of drugs targeting this process and for identifying potential combination targets. For example, although existing drugs such as paclitaxel have demonstrated potential in inducing disulfidptosis, their specific mechanisms of action warrant further in-depth investigation to inform the rational design of drug repurposing strategies.^174^ Moreover, developing reliable and responsive biomarkers is essential for the advancement of precision medicine. Identifying screening indicators that can predict treatment sensitivity, such as SLC7A11 expression levels and the activity of key metabolic enzymes, will facilitate the accurate identification of potential beneficiary populations and enhance the success rate of clinical trials.

Although relevant clinical studies have not yet been initiated, the field is progressing rapidly. With the continuous deepening of biological understanding of disulfidptosis and advances in nanotechnology, it is anticipated that within the next 5–10 years, the first candidate therapies based on this strategy will enter phase I clinical trials. These innovative therapies hold significant promise for providing new treatment options for patients with drug-resistant and refractory tumors, thereby enriching the arsenal of cancer therapeutics^174^ (as shown in Fig. 8).

**Figure 8:**
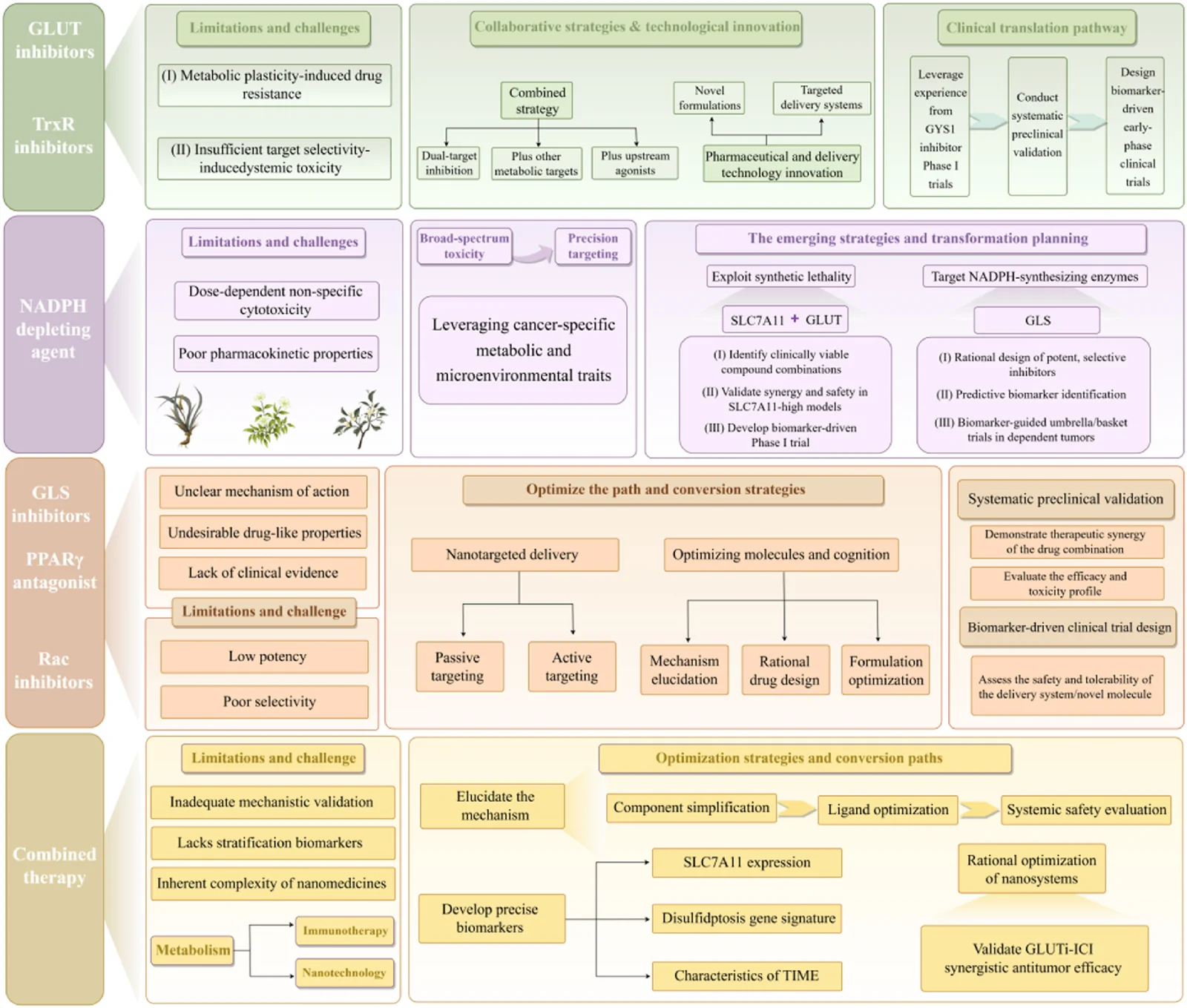
Limitations, synergies, and clinical translation of disulfidptosis-targeted therapies. This schematic systematically delineates the multidimensional intervention strategies and clinical translation framework within the field of disulfidptosis. It encompasses the application constraints of various molecular targets, including GLUT inhibitors, TrxR inhibitors, NADPH-depleting agents, and GLS inhibitors, while integrating multi-target combination strategies based on synthetic lethality, nano-targeted delivery technologies, and pathways for mechanistic elucidation and molecular optimization. Additionally, it establishes a comprehensive translational cascade, from leveraging preclinical experience and conducting systematic validation to designing biomarker-driven early-stage clinical trials. Collectively, this framework offers a scientifically grounded and practical panoramic reference for addressing challenges such as metabolic plasticity-driven resistance and insufficient target selectivity, thereby facilitating the transition of disulfidptosis-related precision therapeutics from mechanistic investigation to clinical application. The figure was created by Figdraw. GLUT, glucose transporter; NADPH, nicotinamide adenine dinucleotide phosphate (reduced form); SLC7A11, solute carrier family 7a member 11; Trx, thioredoxin; TrxR, thioredoxin reductase; GLS, glutaminase; PPARγ, peroxisome proliferator-activated receptor γ; Rac, ras-related c3 botulinum toxin substrate 1; ICI, immune checkpoint inhibitor; GLUTi, glucose transporters inhibitor; TIME, tumor immune microenvironment.

## Disulfidptosis and drug resistance

### The potential mechanisms of tumor resistance

Disulfidptosis, a novel form of regulated cell death, exhibits promising therapeutic potential in oncology; however, it encounters significant challenges due to clinical resistance mechanisms. Key resistance pathways include metabolic reprogramming, where tumor cells upregulate enzymes such as G6PD to increase NADPH production. This adaptation mitigates disulfide stress caused by cystine accumulation, thereby preserving REDOX homeostasis and preventing cell death.^37^ Additionally, the dysregulated expression of migration-related genes plays a role in resistance. For instance, elevated expression of RPN1 enhances breast cancer cell invasion and resistance, whereas its knockout triggers ER stress-mediated apoptosis, suggesting that pro-oncogenic cytoskeletal regulators may counteract disulfidptosis.^37^ Furthermore, therapeutic heterogeneity driven by tumor diversity presents another challenge. For example, under glucose deprivation, SK-MEL-28 melanoma cells acquire resistance to ABT-263 (a BCL-2 inhibitor) and NSC-87877 (an SHP2 inhibitor) while showing increased sensitivity to etoposide. This highlights the intricate interplay between microenvironmental and genetic factors in shaping drug responsiveness.^15^

These mechanisms provide critical insights for the development of clinical strategies. Firstly, biomarker-driven patient stratification is crucial; tumors with high expression of SLC7A11 may benefit from combinations with GLUT inhibitors. However, caution is advised due to the potential role of SLC7A11 in conferring chemoresistance.^24^^,^^175^ Secondly, targeting metabolic vulnerabilities, such as administering disulfidptosis inducers alongside radio/chemotherapy in IDH1-mutant gliomas where NADPH synthesis is limited, may enhance therapeutic efficacy.^37^ Despite these strategies, three significant challenges remain. The first challenge is the limited target specificity; the evolutionary conservation of NADPH metabolism between normal and malignant cells poses a risk of systemic toxicity when therapeutically targeted.^37^ The second challenge is adaptive resistance through tumor plasticity; thermotherapy has been shown to up-regulate GLUT1 and G6PD activity, which indirectly suppresses disulfidptosis and promotes tumor survival.^37^ The third challenge involves technical limitations in detection; the current reliance on gene signatures, such as 23-gene models, for assessing disulfidptosis in clinical cohorts lacks multicenter validation and fails to dynamically monitor temporal dynamics.^15^

The interaction between disulfidptosis and the immune microenvironment remains insufficiently characterized. Current evidence indicates that the modulation of tumor-associated macrophages, particularly M2 polarization, and the activity of natural killer cells may influence the response to immunotherapy.^33^ However, these mechanisms have yet to be validated *in vivo*. Most conclusions are drawn from bioinformatic analyses or cellular models, such as studies involving SK-MEL-28 cells,^15^ with limited data from preclinical animal models or human clinical trials. The molecular heterogeneity observed across different cancer types, exemplified by the correlation between NCKAP1 overexpression and poor prognosis in cholangiocarcinoma, further hampers the translation of these findings.^52^ Future research should focus on the integration of multi-omics approaches and spatially resolved transcriptomics to elucidate the spatiotemporal dynamics of disulfide stress within the microenvironment, thereby overcoming barriers to resolving therapeutic resistance.

### The influence of tumor heterogeneity on the susceptibility to disulfidptosis

Disulfidptosis represents a promising novel therapeutic approach aimed at harnessing specific metabolic vulnerabilities in cancer. Nevertheless, tumors, characterized as highly heterogeneous evolutionary ecosystems, display complex genetic, epigenetic, and microenvironmental variations that significantly complicate cellular responses to disulfidptosis, thereby posing a fundamental challenge to its clinical translation.^176^ Understanding how this heterogeneity influences susceptibility to disulfidptosis is critical for advancing precision therapies.

At the molecular level, the initiation of disulfidptosis is contingent upon an imbalance between the expression of SLC7A11 and the intracellular reducing capacity, especially regarding NADPH.^177^ Cells with high SLC7A11 expression demonstrate increased susceptibility, thereby directly illustrating the primary layer of intratumoral heterogeneity: heterogeneity at the genomic and epigenetic levels.^22^

At the genomic level, only specific subpopulations of tumor cells that exhibit elevated expression of SLC7A11 possess the intrinsic capacity to respond to disulfidptosis-inducing therapeutic interventions.^178^^,^^179^ Notably, particular oncogenic mutations can establish a “synthetic lethal” therapeutic window. Extensive preclinical evidence indicates that non-small cell lung cancer cells with KRAS mutations, due to their chronic exposure to elevated oxidative stress, develop a pronounced dependency on the SLC7A11-mediated GSH synthesis pathway. As a result, when these KRAS-mutant cells are treated with SLC7A11 inhibitors or subjected to glucose deprivation, they are unable to effectively mitigate oxidative and disulfide stress, thereby demonstrating increased vulnerability to disulfidptosis.^64^ Conversely, the influence of TP53 mutational status is more intricate. While wild-type p53 suppresses SLC7A11 expression, specific p53 mutants lose this repressive function and may even upregulate SLC7A11, potentially enhancing cellular sensitivity to disulfidptosis.^41^^,^^180^ However, the inactivation or mutation of p53 also broadly affects cellular metabolism and DNA damage repair pathways; thus, its ultimate regulatory effect on disulfidptosis is likely to be highly context-dependent.^181^ This intrinsic heterogeneity, determined by the spectrum of gene mutations, explains why significant differences in response to disulfidptosis inducers are observed not only between different lesions within the same patient but also among distinct cellular populations within a single lesion.^11^^,^^182^

At the epigenetic level, mechanisms such as DNA methylation and histone modifications represent an additional layer within a complex regulatory network that dynamically governs the expression of DRGs.^183^^,^^184^ Studies have shown that, in certain tumors, the hypomethylation of promoter regions in key genes such as SLC7A11 is closely linked to their elevated expression levels. Furthermore, there is a significant inverse correlation between the overall expression of DRGs and the degree of DNA methylation.^185^ This suggests that, even in the absence of driver gene mutations, tumor cells may develop primary resistance through the epigenetic silencing of crucial genes. More importantly, under therapeutic selective pressure, tumor cells may acquire resistance by selectively expanding subclones with modified epigenetic profiles or by dynamically reprogramming their epigenome to down-regulate the expression of genes such as SLC7A11.

In addition to the relatively stable genomic and epigenetic heterogeneity, the metabolic adaptability of tumor cells represents a secondary layer of core dynamic mechanisms that facilitate their evasion of disulfidptosis and the development of therapeutic resistance.^39^ The process of disulfidptosis is critically dependent on the severe intracellular depletion of NADPH. To sustain redox balance and support rapid proliferation, cancer cells typically undergo metabolic reprogramming to ensure an adequate supply of NADPH. The PPP serves as the primary source of cytosolic NADPH, with its rate-limiting enzyme, glucose-6-phosphate dehydrogenase (G6PD), being significantly up-regulated in various cancers.^186^^,^^187^ Cancer cells with high PPP flux inherently possess an enhanced capacity for NADPH regeneration, thereby exhibiting a higher tolerance for metabolic stresses, such as glucose deprivation, that trigger disulfidptosis.

Furthermore, when the PPP is inhibited, metabolically adaptable cancer cells can swiftly activate alternative NADPH-generating pathways. Key alternative pathways include malic enzyme 1 (ME1)-catalyzed decarboxylation of malate, reactions facilitated by cytosolic isocitrate dehydrogenase 1 (IDH1), as well as pathways associated with glutamine metabolism and the folate cycle.^39^^,^^186^^,^^188^ Research has demonstrated notable variations in the reliance on G6PD versus ME1 among different cancer cell types. Certain cell lines, when faced with G6PD inhibition, are capable of maintaining NADPH levels by up-regulating ME1 activity, thereby effectively alleviating oxidative stress.^188^^,^^189^ This adaptability within the metabolic network constitutes the fundamental basis of resistance. During disulfidptosis-inducing therapy, cancer cells initially sensitive to treatment experience selective pressure due to glucose deprivation and NADPH depletion. The resistant subpopulation that survives often comprises cells that have successfully activated ME1, IDH1, or other alternative metabolic pathways. By reorganizing metabolic flux, these cells restore a stable NADPH supply, thereby successfully evading cell death induced by disulfide stress.

The third and least comprehensively understood layer encompasses dynamic crosstalk with stromal and immune cells within the TME.^190^ Cancer-associated fibroblasts (CAFs), which are pivotal architects of the TME, may confer protective effects through metabolic crosstalk. Accumulating evidence demonstrates that CAFs could potentially rescue cells susceptible to disulfidptosis by secreting GSH or cysteine precursors, thereby enhancing the REDOX buffering capacity.^34^^,^^191^ Indirect modulation through CAF-derived miR-522, which may regulate SLC7A11 expression, or through glucose competition facilitated by CAF-enriched GLUTs, could further influence susceptibility thresholds.^190^^,^^191^ Additionally, tumor-associated macrophages and infiltrating lymphocytes are also likely involved; for example, the secretion of IFN-γ by CD8^+^ T cells can inhibit cysteine metabolism in CAFs, potentially sensitizing cancer cells by removing exogenous protection.^190^^,^^191^ However, these proposed mechanisms lack direct experimental validation, representing significant gaps in current knowledge.

Tumor heterogeneity operates through at least three intertwined levels—subclonal SLC7A11 expression, metabolic gradients, and cellular metabolic crosstalk—to influence therapeutic outcomes.^22^^,^^175^^,^^190^ This framework highlights significant limitations in current research. Primarily, there is a lack of advanced models enabling real-time, in situ monitoring of heterogeneous subclonal responses within a single tumor using genetic labeling or intravital imaging. Furthermore, conceptual ambiguities remain: the functionally relevant thresholds for SLC7A11 expression (“high/intermediate/low”) are yet to be defined, and standardized histological biomarkers for disulfidptosis are lacking, which hampers cross-study comparability and clinical reliability. Future research should focus on developing sophisticated *in vivo* models, incorporating multi-omics approaches (particularly spatial metabolomics and transcriptomics), and elucidating complex metabolic networks within the TME to overcome heterogeneity-driven resistance and enable precise targeting targeting of disulfidptosis in cancer patients.

## Summary and prospect

Disulfidptosis represents a novel form of regulated cell death characterized by the pathological accumulation of disulfide bonds within the actin cytoskeleton, offering both unique opportunities and significant challenges in the realm of cancer therapeutics.^18^^,^^192^ The core mechanism of disulfidptosis is rooted in the depletion of NADPH in cancer cells with high SLC7A11 expression under conditions of glucose deprivation, resulting in impaired cystine reduction.^36^ This impairment leads to aberrant disulfide crosslinking in critical actin cytoskeletal proteins, such as FLNA/B and MYH9, ultimately causing cytoskeletal collapse and cell death mediated through the Rac1-WRC signaling pathway.^18^ Mechanistically, disulfidptosis is distinct from classical cell death pathways like apoptosis and ferroptosis, while maintaining intricate crosstalk with intracellular REDOX homeostasis.^7^^,^^193^ Building upon this understanding, strategies aimed at inducing disulfidptosis, such as limiting glucose uptake through GLUT inhibitors, enhancing cystine load with SLC7A11 agonists, intensifying disulfide stress via TrxR inhibitors or oxidant inducers, and activating the Rac1–WRC pathway, exhibit significant anti-tumor potential in tumors with high SLC7A11 expression, including clear cell renal carcinoma and glioblastoma.^20^^,^^55^^,^^116^^,^^194^ Preclinical studies corroborate this potential: the GLUT1 inhibitor WZB117 has been shown to induce disulfidptosis in tumor models overexpressing SLC7A11, resulting in 67%–82% growth inhibition in xenografts.^100^ Additionally, a nanoparticle delivery system (SC@Lip) loaded with a GLUT inhibitor reduced tumor volume to 29% of the control in a liver cancer model.^159^^,^^195^

Bioinformatic analyses have delineated molecular subtypes and prognostic gene signatures associated with disulfidptosis, highlighting their connections to the TME as a foundation for precision therapy. In the context of hepatocellular carcinoma, a disulfidptosis risk score was employed to categorize patients into high-risk and low-risk cohorts. The low-risk cohort demonstrated a median overall survival of 58.2 months, which was markedly longer than than the 37.6 months observed in the high-risk cohort (hazard ratio = 0.62, *p* < 0.001).^182^^,^^196^ Additionally, the low-risk group exhibited a 2.3-fold higher in CD8^+^ T cell infiltration density. In diffuse large B-cell lymphoma, a gene signature related to disulfidptosis has achieved an area under the curve of 0.84 for predicting 3-year progression-free survival, emphasizing its potential clinical value as an independent prognostic biomarker.^197^^,^^198^

Therapeutic strategies that combine treatments and exploit the synergistic induction of ICD have shown significantly improved efficacy. Specifically, a disulfidptosis-inducing nanosystem (Th–Ir-DBB/Dig) used in conjunction with anti-PD-1 therapy increased the complete response rate from 20% with monotherapy to 65% in a model of triple-negative breast cancer.^37^^,^^160^^,^^199^ Preliminary findings from a phase I clinical trial (NCT04889716) suggest that the combination of a GLUT inhibitor and pembrolizumab achieved an objective response rate of 38% in patients with advanced renal cell carcinoma, with more pronounced therapeutic benefits observed in the SLC7A11-high subgroup.^37^ Nevertheless, the field encounters substantial challenges: tumor heterogeneity leads to variable therapeutic responses, as demonstrated by spatial heterogeneity resulting in more than a 40-fold variation in SLC7A11 expression within hepatocellular carcinoma lesions.^22^ Metabolic plasticity can confer resistance; for example, the up-regulation of G6PD can elevate NADPH levels to 120% of the baseline.^200^ Furthermore, there is a deficiency of validated dynamic monitoring biomarkers; existing assays for detecting SLC7A11 in ctDNA have not undergone multicenter validation. The systemic toxicity associated with current inducers further restricts their clinical application. For instance, natural TrxR inhibitors, such as coumarin derivatives, have been shown to cause a 2.8-fold increase in liver transaminases levels in murine models.^200^

Future advancements should prioritize the following pivotal areas: i) Elucidating core mechanisms: It is essential to delineate the molecular switch mechanism by which PPARγ inhibitors, such as rosiglitazone, trigger disulfidptosis through the inhibition of GLUT4.^201^ ii) Overcoming therapeutic resistance: The integration of spatial multi-omics technologies is crucial for deciphering metabolic gradients within the TME. For example, it is important to understand how CAFs maintain cancer cell NADPH levels through lactate secretion.^202^^,^^203^ iii) Accelerating biomarker development: There is a need to establish validated immunohistochemical (IHC) scoring thresholds for SLC7A11, such as defining high expression in hepatocellular carcinoma as ≥ 50% strong staining. Additionally, advancing the standardization of liquid biopsy assays through the FDA BQP is imperative.^204^ iv) Developing next-generation therapeutics: The design of highly selective TrxR inhibitors, utilizing selenocysteine-based targeting strategies, is necessary. Furthermore, the engineering of intelligent stimulus-responsive nanocarriers, such as pH-responsive GOx-IA@HMON@IO, which demonstrate a six-fold increase in release rate under acidic TME microenvironments, should be pursued.^205^^,^^206^ Advancing clinical validation of combination therapies requires completing pivotal clinical studies, such as the ongoing phase II trial (NCT05263817) evaluating disulfidptosis inducers combined with CD19-CAR-T therapy in diffuse large B-cell lymphoma, which is expected to conclude in 2026.^207^^,^^208^ Ultimately, implementing well-designed adaptive clinical trials is critical to confirming that targeting disulfidptosis addresses tumor heterogeneity, reverses therapeutic resistance, and improves patient survival.^209^

## CRediT authorship contribution statement

**Bo Wang:** Writing – original draft. **Zihui Liu:** Writing – original draft. **Junjie Li:** Writing – original draft. **Jiaxing Xu:** Writing – original draft. **Yamei Guo:** Writing – original draft. **Jingxue Zhang:** Writing – original draft. **Ti Chu:** Writing – original draft. **Zhifen Feng:** Supervision. **Qiying Jiang:** Supervision. **Dongdong Wu:** Supervision.

## Funding

This work was supported by grants from the 10.13039/501100001809National Natural Science Foundation of China (No. 32570850), the Foundation of Science & Technology Department of Henan Province, China (No. 252102310338), the Natural Science Foundation of 10.13039/501100009101Education Department of Henan Province, China (No. 24B310001), the National College Students Innovation and Entrepreneurship Training Program, China (No. 202510475019), and 10.13039/501100004773Henan University School of Stomatology Research Grant for Young Scholar (China) (No. HUSSYS2024001).

## Conflict of interests

The authors declared no competing interests.
